# Supplementary figures and images for: An interbacterial cysteine protease toxin inhibits cell growth by targeting type II DNA topoisomerases GyrB and ParE
Source: PLoS Biol. 2025 May 27;23(5):e3003208. doi: 10.1371/journal.pbio.3003208 (PMC12136450; doi:10.1371/journal.pbio.3003208)

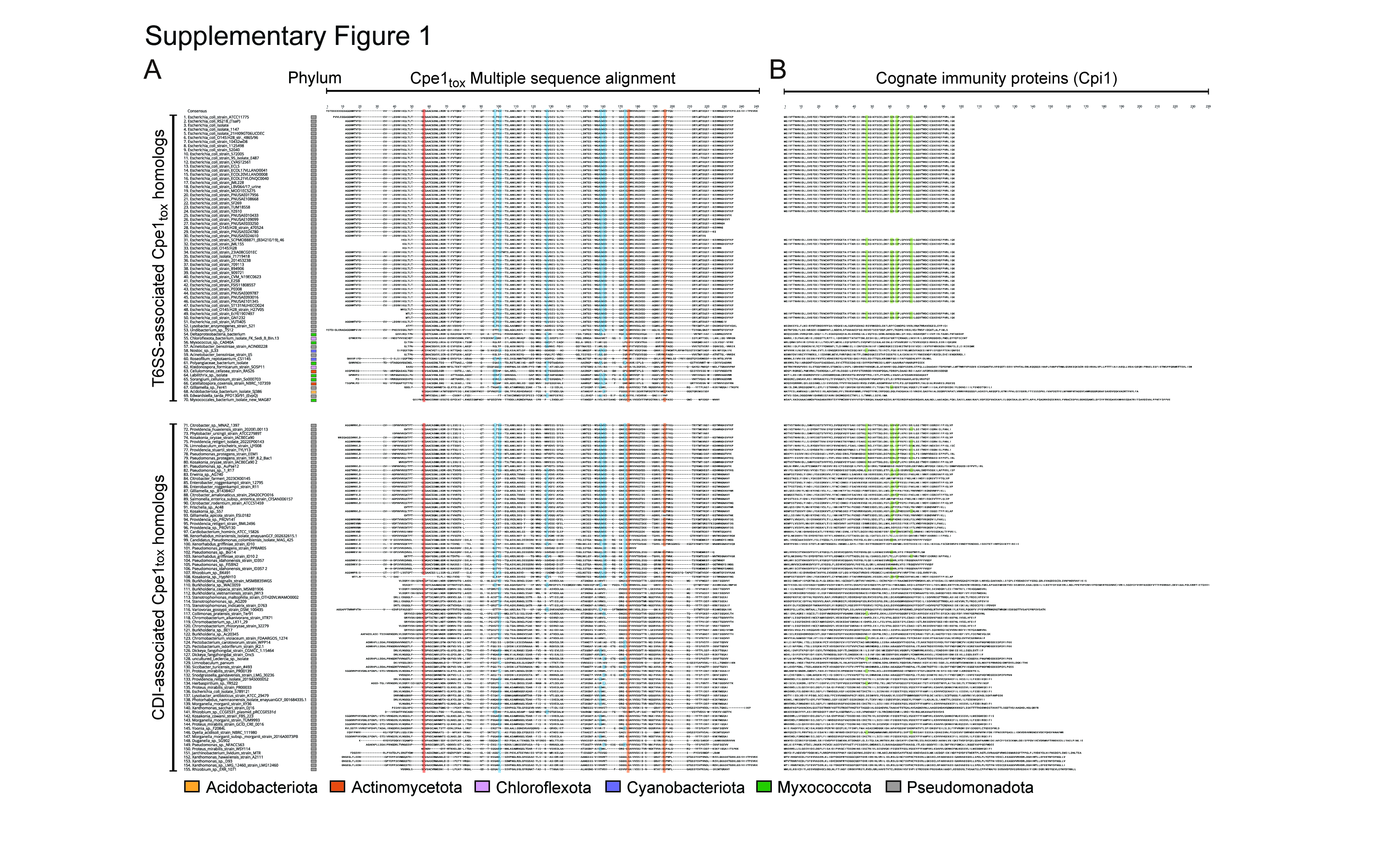

Supplement: S1 Fig — Related to Fig 1b. (a) Multiple sequence alignment of 155 unique sequences of interbacterial PLCP effectors. Sequences have been ordered by relative identity to Cpe1tox of E. coli ATCC-11775 and are displayed in two clusters divided according to the associated secretion systems (T6SS or CDI). Conserved residues are indicated as follows: the catalytic triad residues are highlighted by red (Cysteine) and orange (Histidine and Aspartate/Asparagine) shadows; and hydrophobic residues composing the surface concave region are highlighted by blue shadows. The Phylum of each strain containing the interbacterial PLCP effectors is indicated by colored boxes (as indicated in the legend). (b) Sequences of the putative cognate immunity proteins related to the interbacterial PLCP effectors in (a). Conserved binding residues are highlighted in green shadows. Blank rows indicate an absence of putative immunity proteins due to incomplete genomic data for certain bacterial species in the NCBI database. See also S1 Table for detailed information. (TIF) [file pbio.3003208.s001.tif]

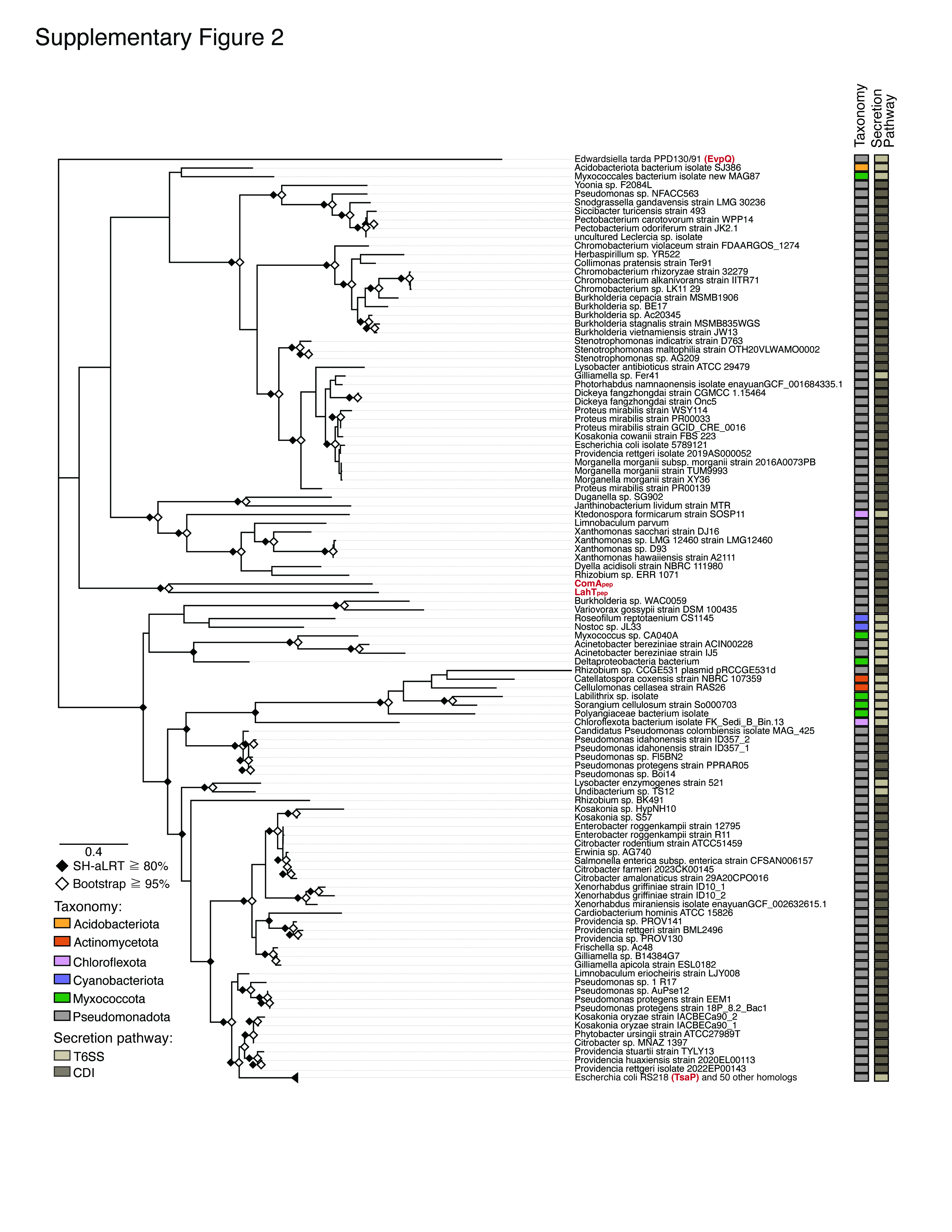

Supplement: S2 Fig — Related to Fig 1d. Maximum likelihood phylogeny of 155 unique PLCP effector sequences visualized as a rectangle tree. Branch support values (SH-aLRT and ultrafast bootstrap) are displayed as symbols (filled diamond: SH-aLRT ≥ 80%, hollow diamonds: ultrafast bootstrap ≥ 95%). Tips representing sequences of the closely related housekeeping PLCPs, ComApep and LahTpep, as well as the previously identified interbacterial PLCP effectors EvpQ and TsaP, are highlighted in red. The taxonomy of strains containing the PLCP effectors and their associated secretion pathways is indicated on the right as colored boxes. The scale bar represents the average number of substitutions per site. See also S1 Table for detailed information. The original tree file used to generate this figure is available as S2 Data. (TIF) [file pbio.3003208.s002.tif]

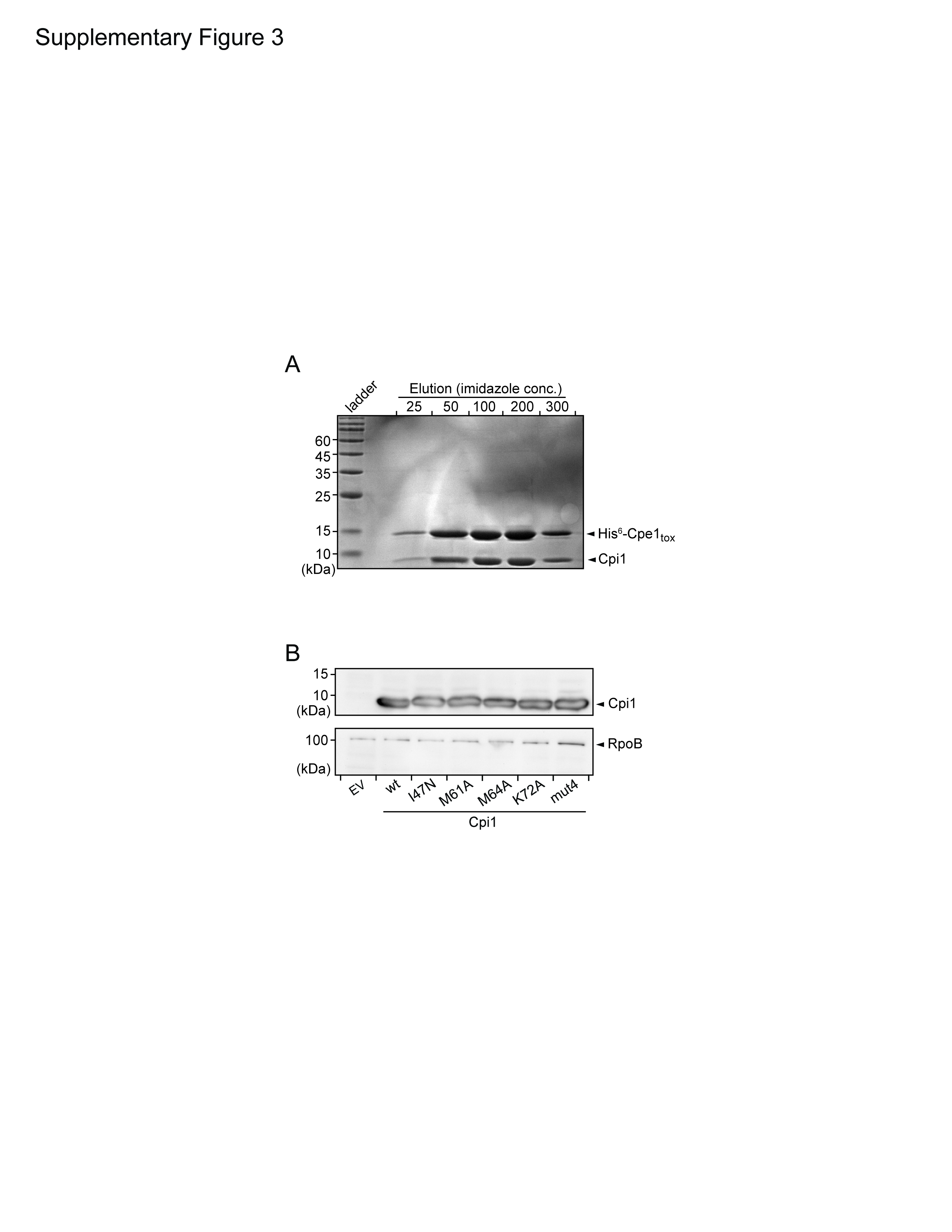

Supplement: S3 Fig — Related to Fig 2. (a) Coomassie-stained SDS–PAGE analysis of the His-tagged Cpe1tox co-purified with Cpi1. (b) Protein expression levels of wild-type Cpi1 and variants, as assessed by immunoblotting analysis. The cytosolic protein RpoB was used as a loading control. The original images are available in S1 Raw Images. (TIF) [file pbio.3003208.s003.tif]

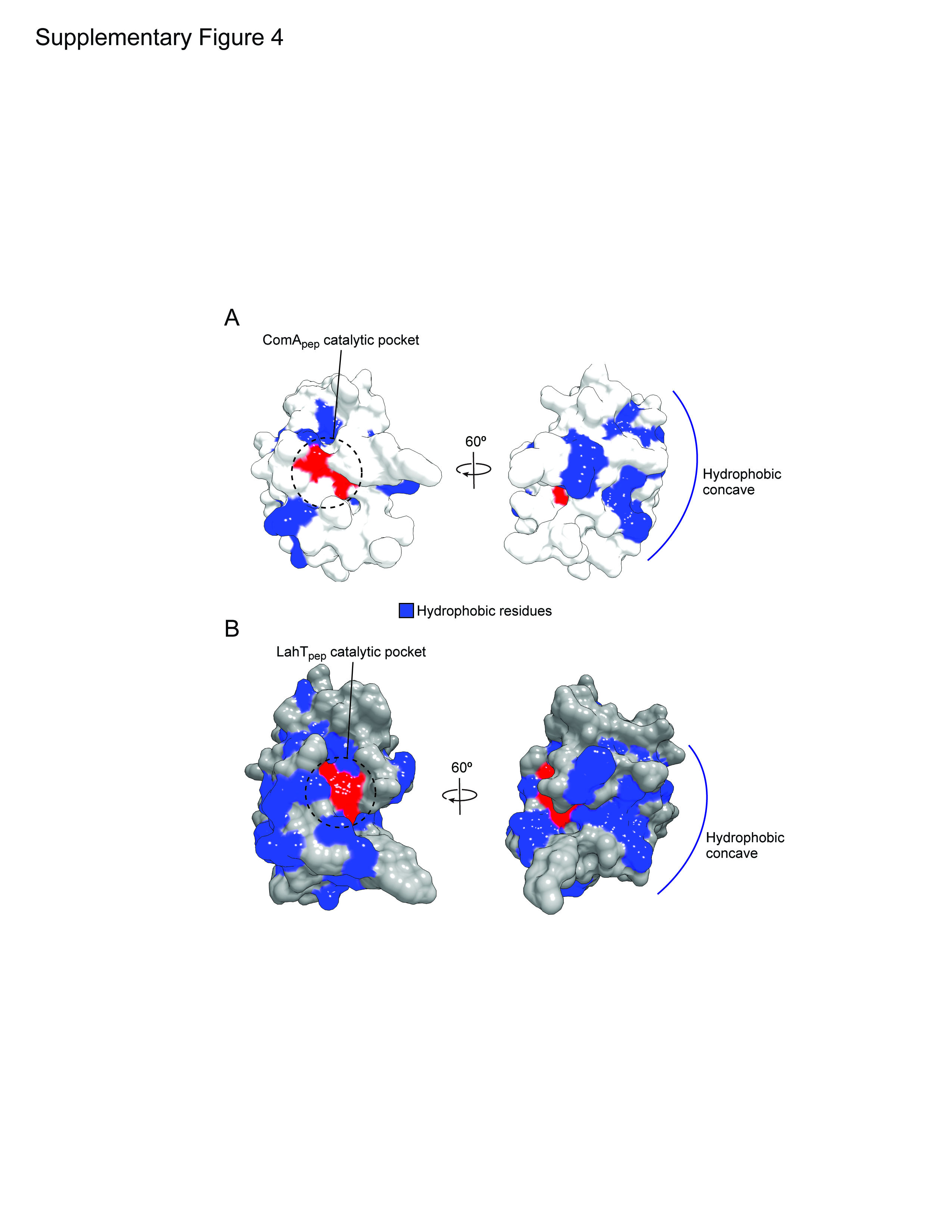

Supplement: S4 Fig — Related to Fig 3. (a, b) Depiction of ComApep (a) and LahTpep (b) illustrating the location of their respective catalytic pockets and hydrophobic concave surfaces. ComApep (PDB: 3K8U, light gray). LahTpep (PDB: 6MPZ, dark gray). (TIF) [file pbio.3003208.s004.tif]

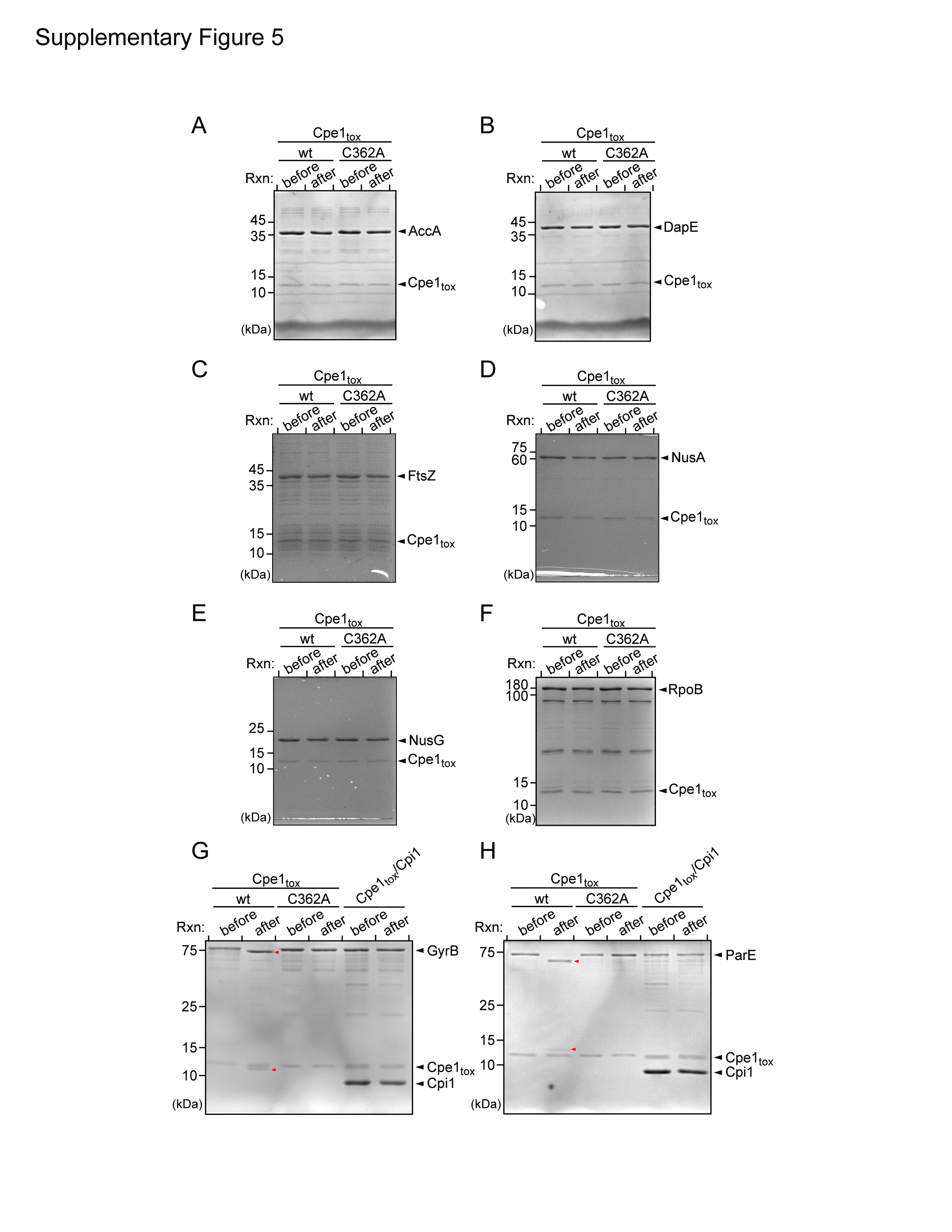

Supplement: S5 Fig — Related to Fig 3. (a-f) Coomassie-stained SDS–PAGE analysis of the in vitro cleavage assay of six candidate substrates of Cpe1tox: AccA (a), DapE (b), FtsZ (c), NusA (d), NusG (e), and RpoB (f). (g, h) Presence of Cpi1 suppressed the cleavage of GyrB (g) and ParE (h) by Cpe1tox. The substrates were incubated with Cpe1tox (lanes 1 and 2), Cpe1toxC362A (lanes 3 and 4), or Cpe1tox and Cpi1 (lanes 5 and 6). Cleaved fragments are indicated with red arrowheads. The original images are available in S1 Raw Images. (TIF) [file pbio.3003208.s005.tif]

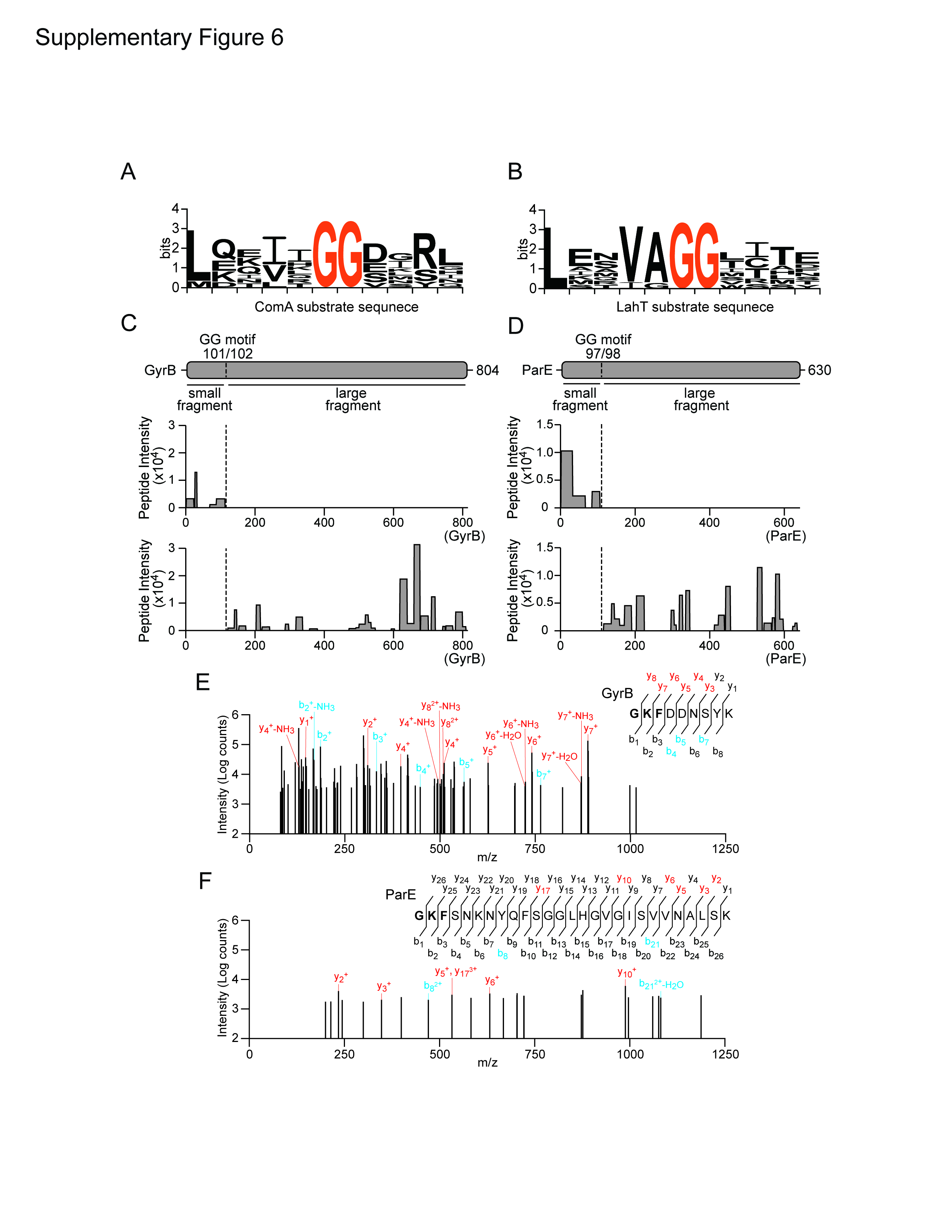

Supplement: S6 Fig — Related to Fig 4. (a, b) Sequence logos showing the consensus sequence recognized by ComA (a) and LahT (b). (c, d) Mapping of peptide sequences from Cpe1tox-digested GyrB (c) and ParE (d) fragments. Cpe1tox-cleaved fragments, resolved by Coomassie-stained SDS–PAGE, were purified, trypsin-digested, and subjected to MALDI-TOF analysis. The intensity of the signals (Y-axis) from identified peptides and their coverage across the respective full-length protein (X-axis) are plotted below. Upper chart: peptides from the small fragment. Lower chart: peptides from the large fragment. (e, f) Tandem mass spectrum of indicated peptides from Cpe1tox-cleaved GyrB (e) and ParE (g) fragments. Fragmentation ions (b, blue; y, red) with resolved spectra and the residues correlating to the LHAGGKF motif (bold) are indicated. The quantitative data for generating plots can be found in S1 Data. The raw proteomics data can be found in https://doi.org/10.5281/zenodo.15361709. (TIF) [file pbio.3003208.s006.tif]

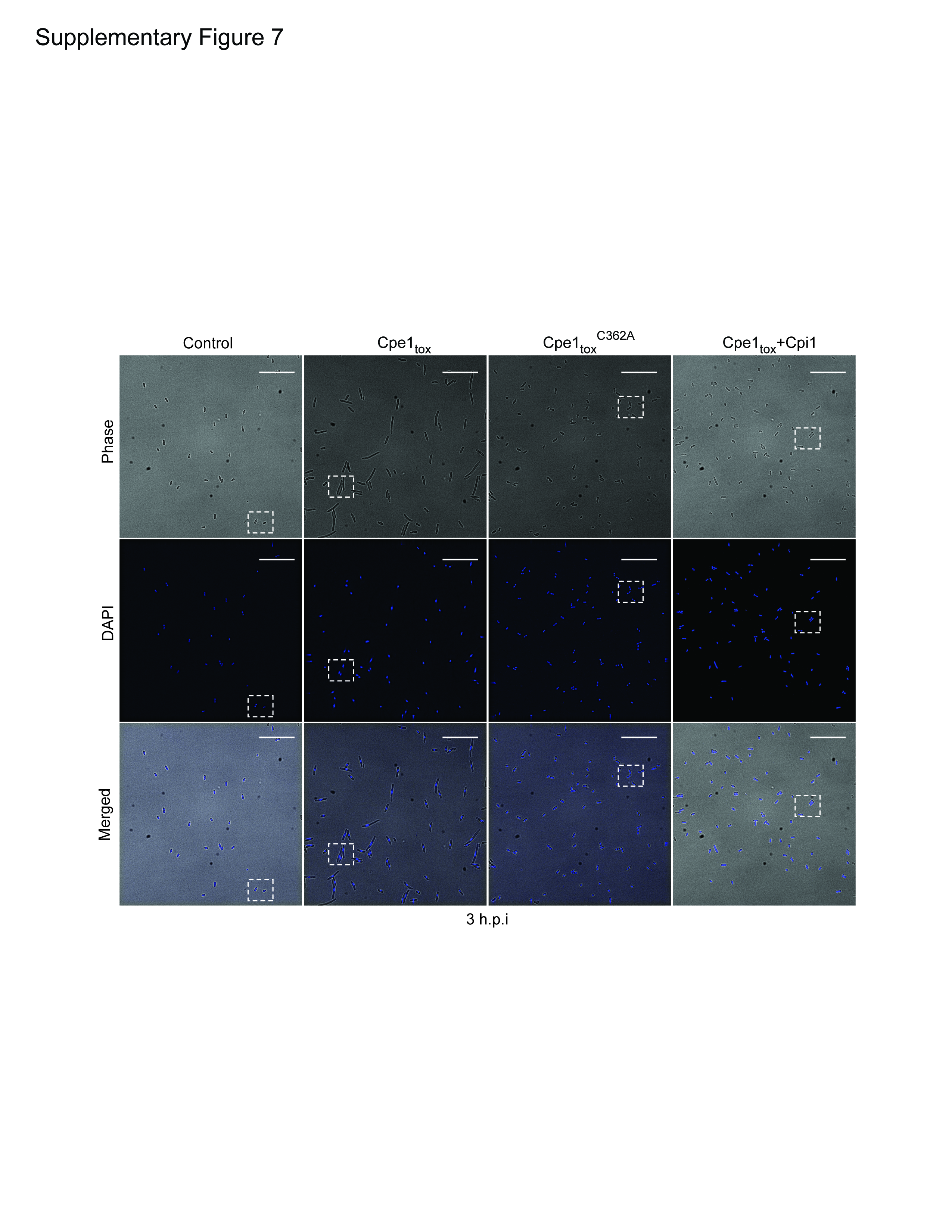

Supplement: S7 Fig — Related to Fig 4f. (a) Phase-contrast (top), blue fluorescence (middle), and merged (bottom) images of E. coli carrying an empty vector, E. coli expressing Cpe1tox, E. coli expressing Cpe1toxC362A, or E. coli co-expressing Cpe1tox and Cpi1, after three hours of induction. Scale bar = 20 μm. The white borders demarcate the cropped images displayed in Fig 4f. (TIF) [file pbio.3003208.s007.tif]

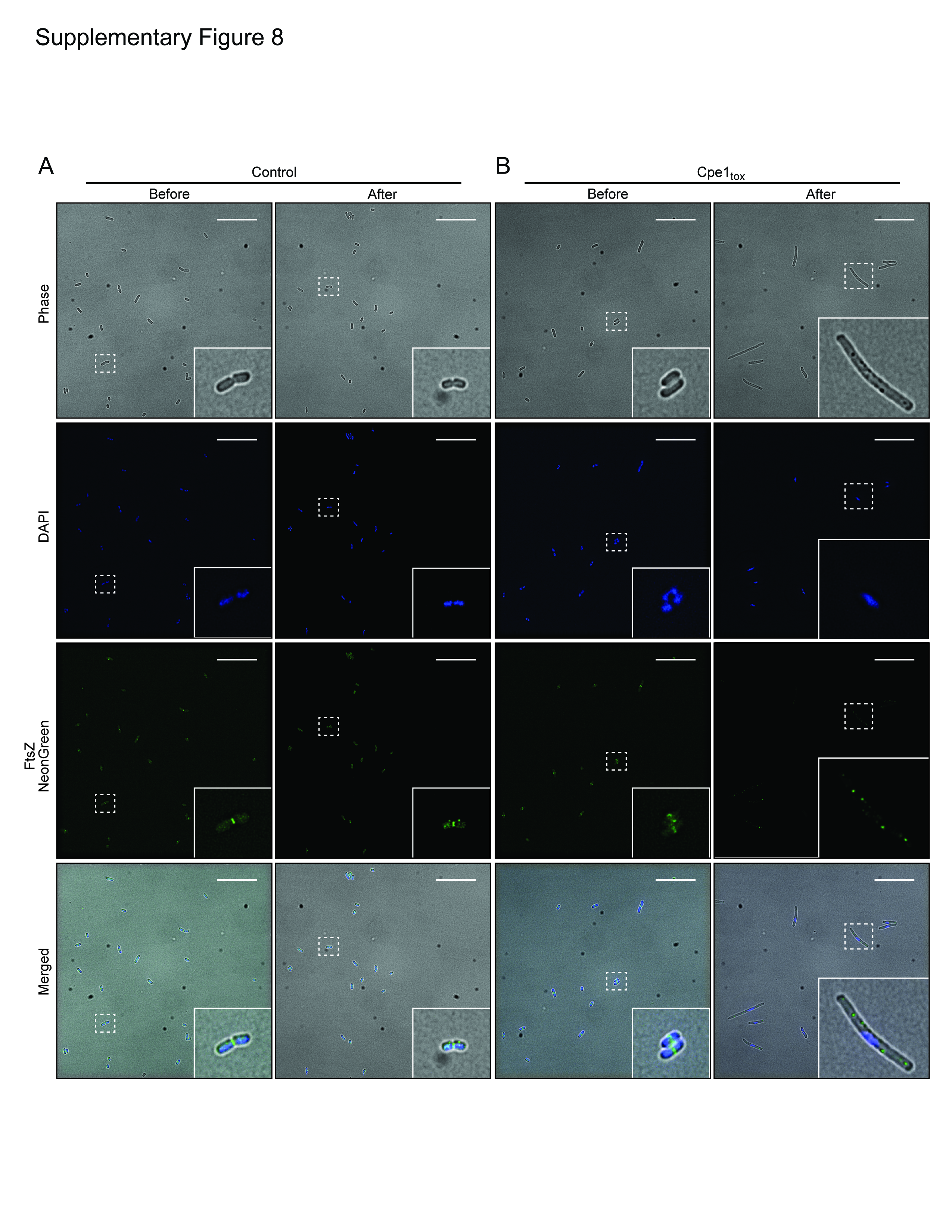

Supplement: S8 Fig — Related to Fig 4f. (a) Phase-contrast (top), blue fluorescence (second from top), green fluorescence (third from top), and merged (bottom) images of E. coli carrying an empty vector, shown before and after 2-h incubation. (b) Fluorescence micrographs of E. coli intoxicated by Cpe1tox. Phase-contrast (top), blue fluorescence (second from top), green fluorescence (third from top), and merged (bottom) images are presented. White borders indicate the zoomed-in regions shown in the bottom-right corner of each image. Scale bar = 20 μm. (TIF) [file pbio.3003208.s008.tif]

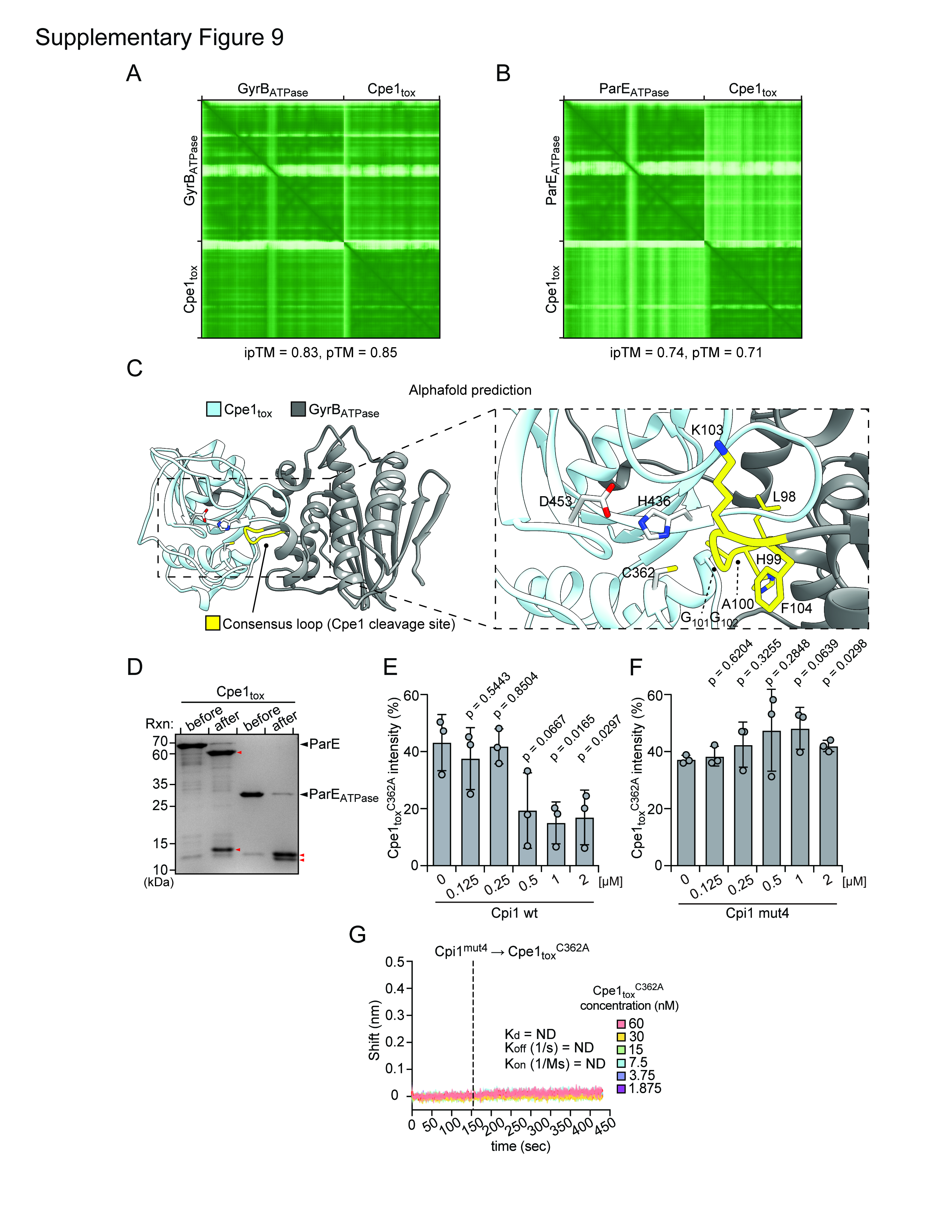

Supplement: S9 Fig — Related to Fig 5a, 5c, 5d, 5e, 5f. (a, b) Predicted assigned error (PAE) plots for the models of the Cpe1tox–GyrBATPase interaction (a) and the Cpe1tox–ParEATPase interaction (b) in Fig 5a. Accuracy of the predicted relative positions of subunits within the complex, as indicated by AlphaFold 3 prediction scores (iPTM). Confidence in the overall folding of the complex is indicated by pTM scores. (c) Predicted structure of Cpe1tox–GyrBATPase interaction, showing the entrance of the consensus loop of GyrB into the active site of Cpe1 (left panel). The magnified view of the active pocket (right panel) implies possible catalysis. (d) Cpe1tox targets the ATPase domain of ParE. Cleavage of full-length ParE and the ATPase domain by Cpe1tox was analyzed by Coomassie-stained SDS–PAGE. Cleaved fragments are indicated with red arrowheads. (e, f) Quantifications of the results of the competitive binding assay shown in Fig 5c (e) and 5d (f). Results from three independent assays were plotted on a column graph as mean ± SD. The Y-axis represents levels of Cpe1tox normalized to the level of ParEATPase on the same lane (%). The X-axis represents the concentration of wild-type or mutant Cpi1 in the reaction. P-values were calculated using Student t test to compare each result from that of no competition binding (0 μM Cpi1). (g) Results of kinetic assays on interactions between Cpi1mut4 and Cpe1toxC362A using biolayer interferometry (BLI). Data from three replicates have been plotted on an X–Y scatter graph. The Y-axis represents wavelength shifts (nm) generated by the binding of the two proteins, which are nearly non-detectable (N.D.). The X-axis represents reaction time in seconds. A vertical dashed line marks the transition from the association step to the dissociation step. Kinetic parameters such as Kd, Kon, Koff, and Rmax could not be calculated and are labeled as ND. The data underlying this figure are available in S1 Data and S1 Raw Images. (TIF) [file pbio.3003208.s009.tif]

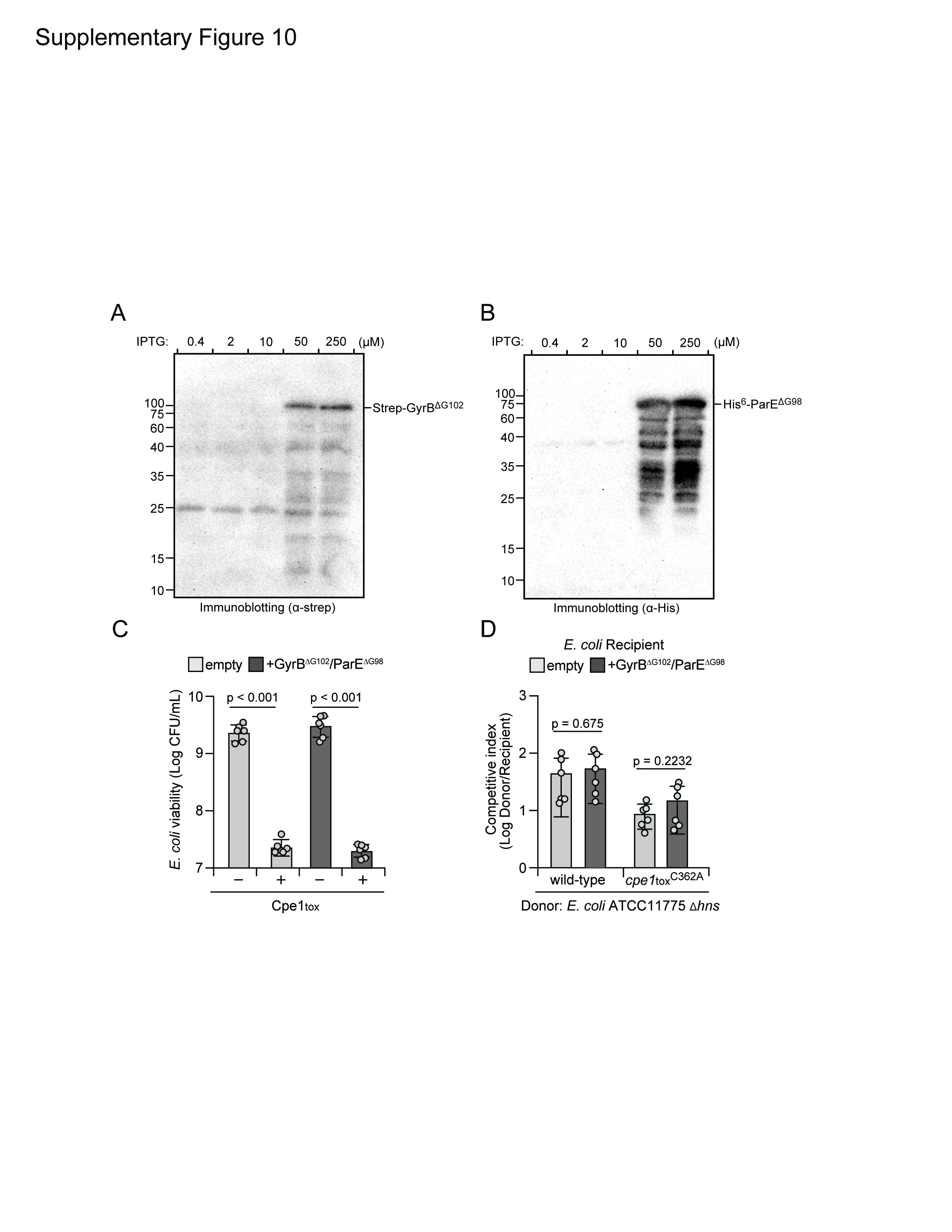

Supplement: S10 Fig — (a, b) Expression level of Strep-tagged GyrBΔG102 (a) and His-tagged ParEΔG98 (b) in E. coli susceptible to Cpe1. (c) Viable cells recovered from plating cultures carrying plasmids expressing the indicated proteins. E. coli carrying plasmid-borne GyrBΔG102 and ParEΔG98 or an empty vector was included to validate suppression of Cpe1 toxicity. (d) Bacterial competition assays were performed between the indicated donor cell carrying Cpe1 and the susceptible strain. Experiments were conducted on a solid agar supplemented with inducing agents to maintain expression of plasmid-borne GyrBΔG102 and ParEΔG98 in the recipient strain. The data in (c) and (d) are shown as the mean ± SD; n = 6. Both data are representative experiments out of at least 3 independent experiments. P values were calculated with Student t test to assess differences in viability among populations (c), and to evaluate statistically significant differences in the competitive indices of each donor strain against the specified recipients (d). The data underlying this figure are available in S1 Data and S1 Raw Images. (TIF) [file pbio.3003208.s010.tif]
