# Supplementary material for: An interbacterial cysteine protease toxin inhibits cell growth by targeting type II DNA topoisomerases GyrB and ParE
Source: PLoS Biol. 2025 May 27;23(5):e3003208. doi: 10.1371/journal.pbio.3003208 (PMC12136450; doi:10.1371/journal.pbio.3003208)

Figure 2

Figure 2B

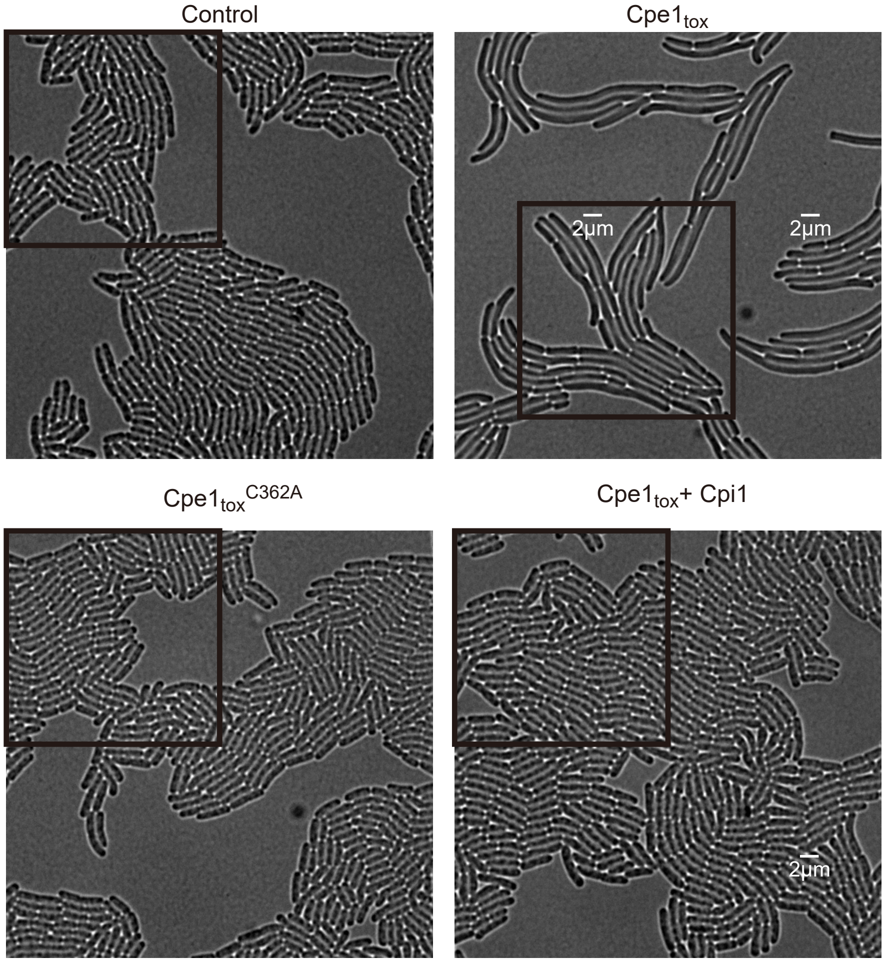

Figure 2I

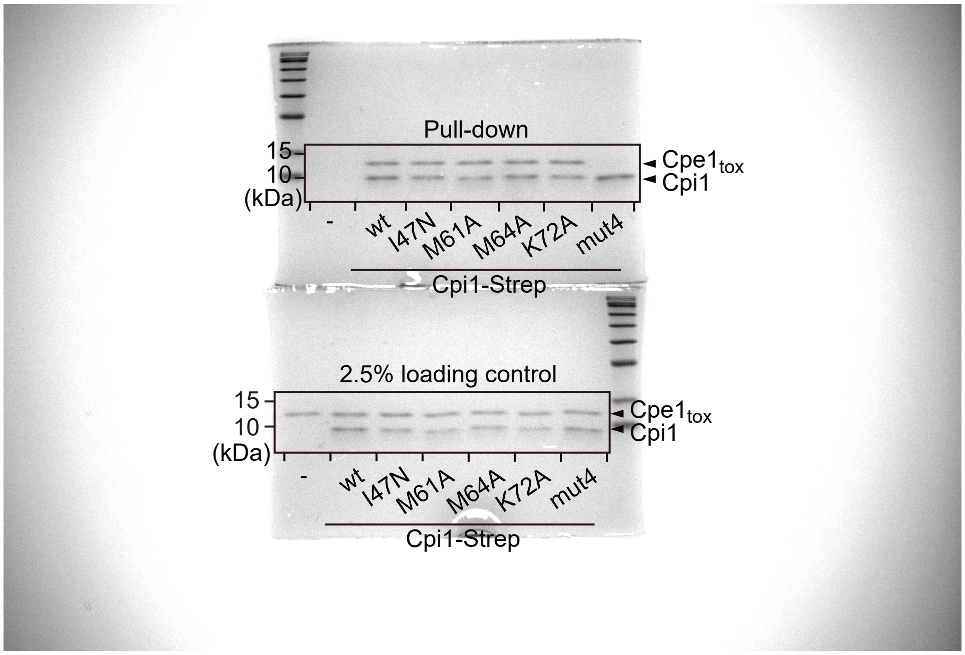

Figure 3

Figure 3A

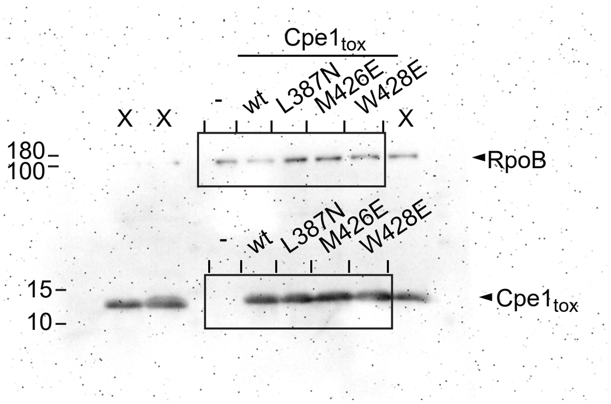

Figure 3C

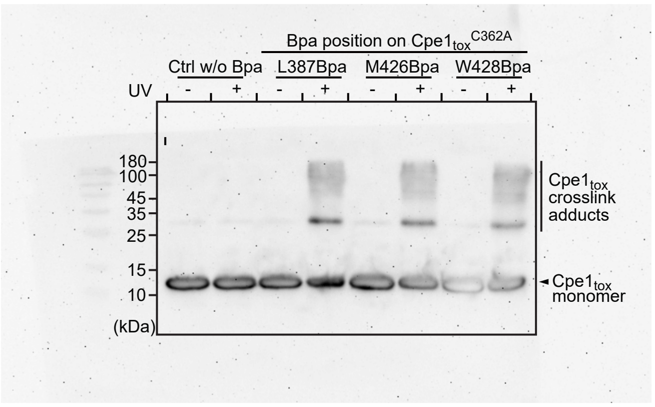

Figure 3E

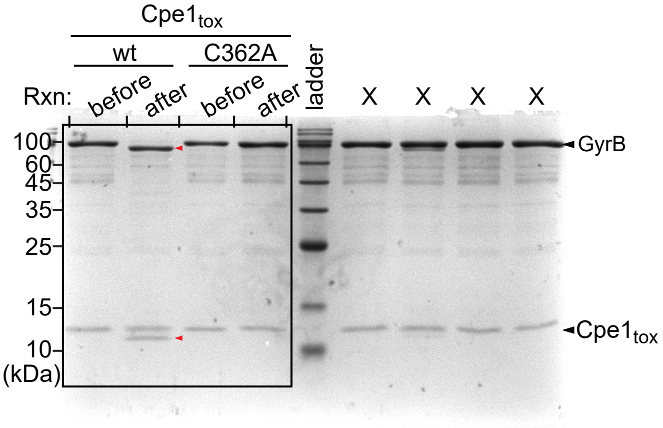

Figure 3F

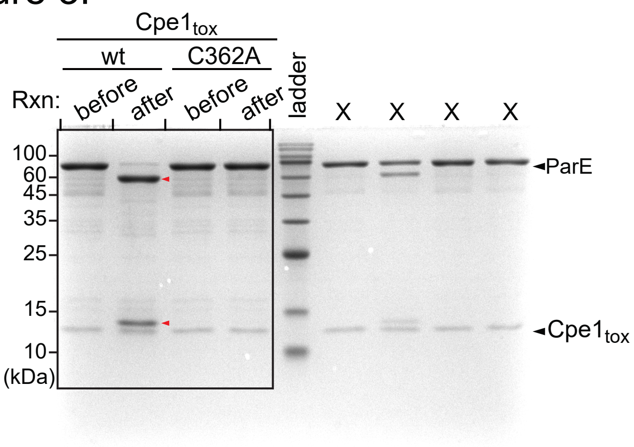

Figure 3H

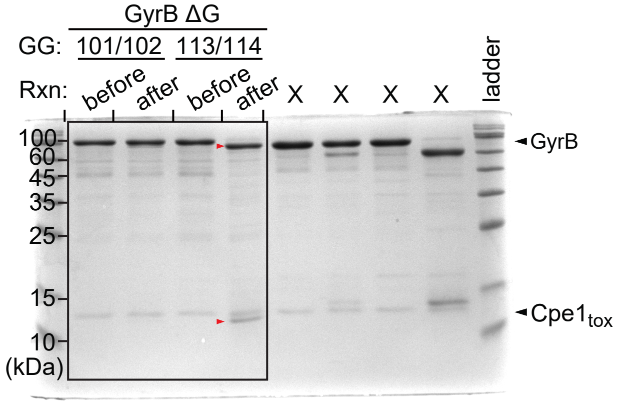

Figure 3I

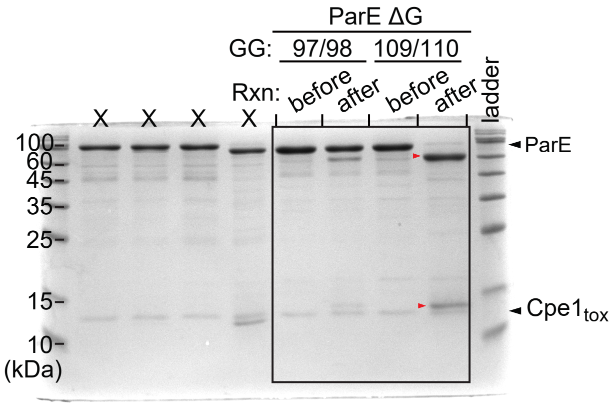

Figure 4

Figure 4E

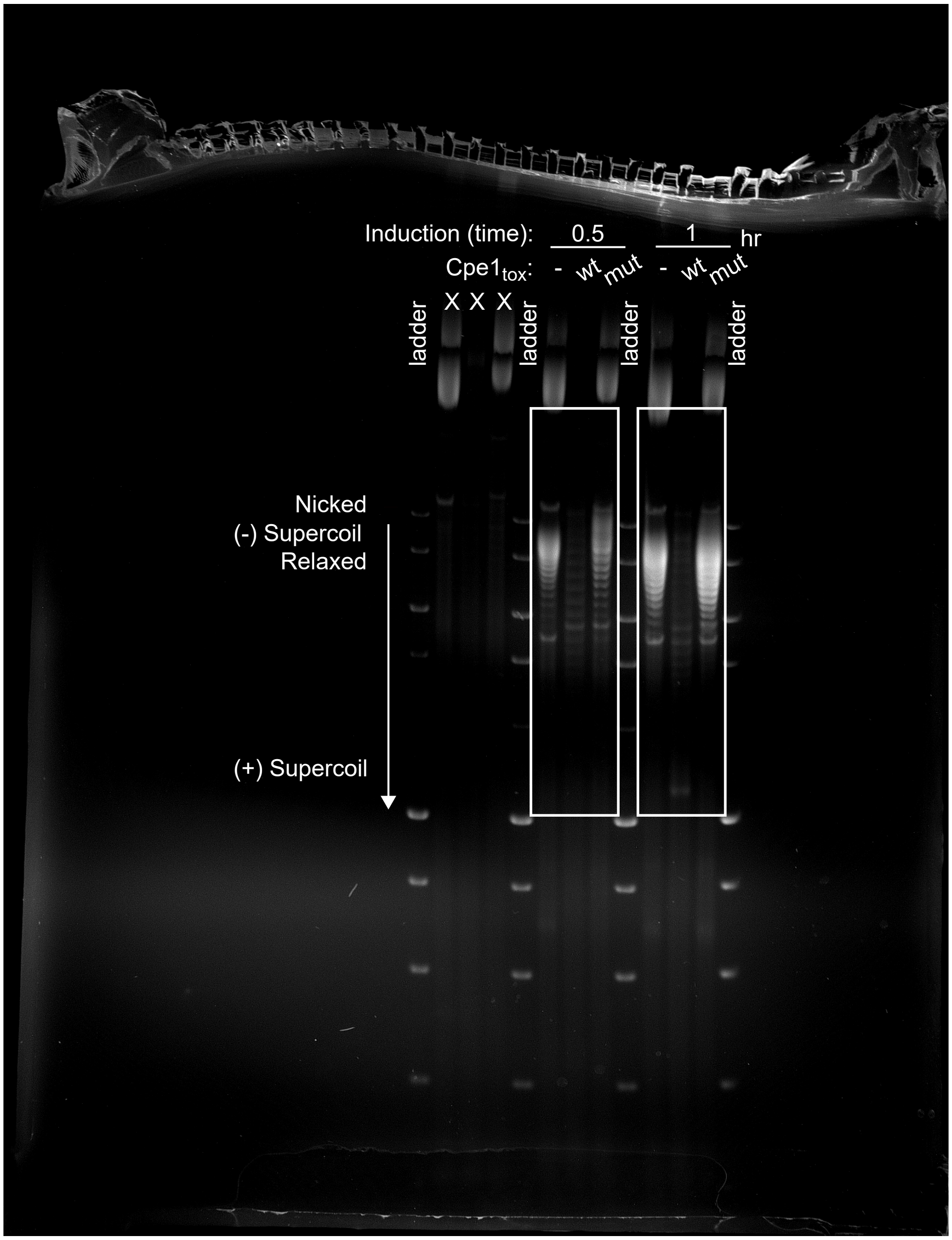

Figure 5

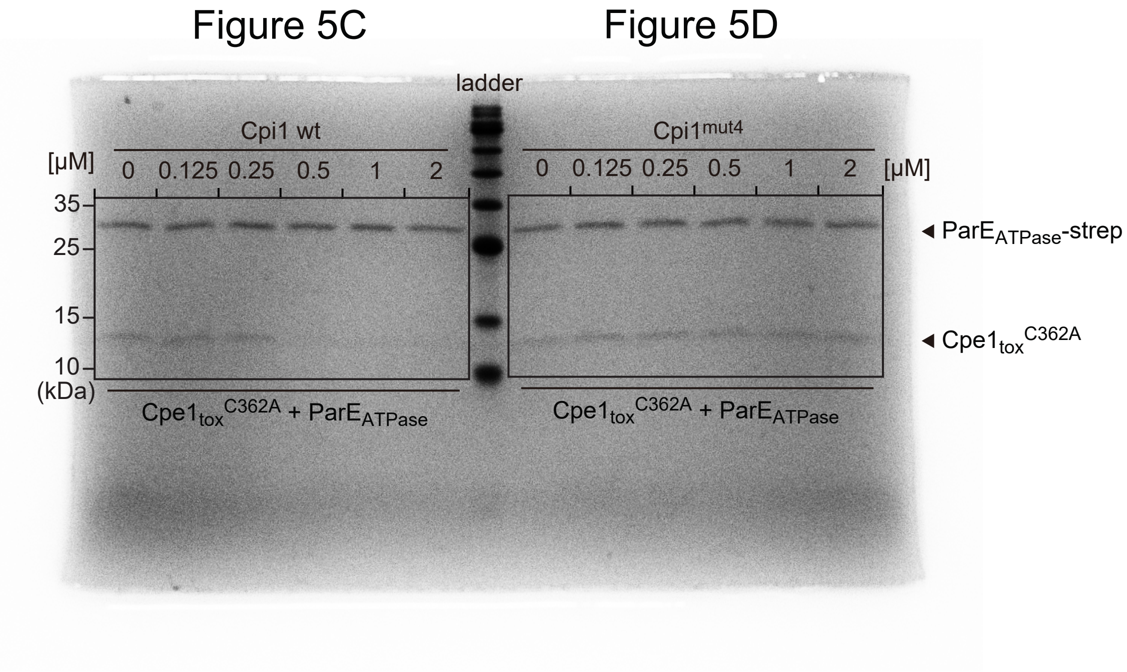
Figure 5C
Figure 5D
ladder
Cpi1 wt
Cpi1mut4
[μM]
0 0.125 0.25 0.5 1 2
0 0.125 0.25 0.5 1 2
[μM]
35
25
15
10
(kDa)
ParEATPase-strep
Cpe1toxC362A
Cpe1toxC362A + ParEATPase
Cpe1toxC362A + ParEATPase

Supplementary Figure 3

Supplementary Figure 3A

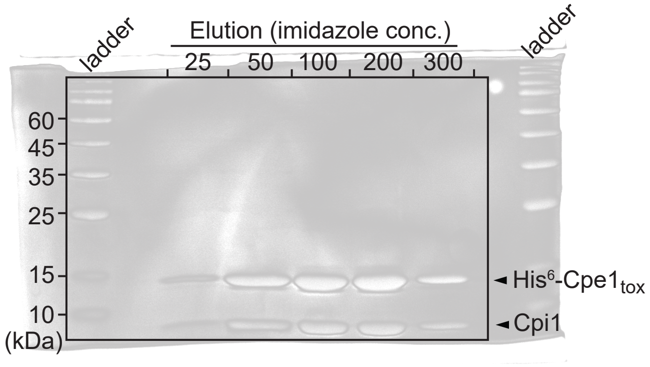

Supplementary Figure 3B

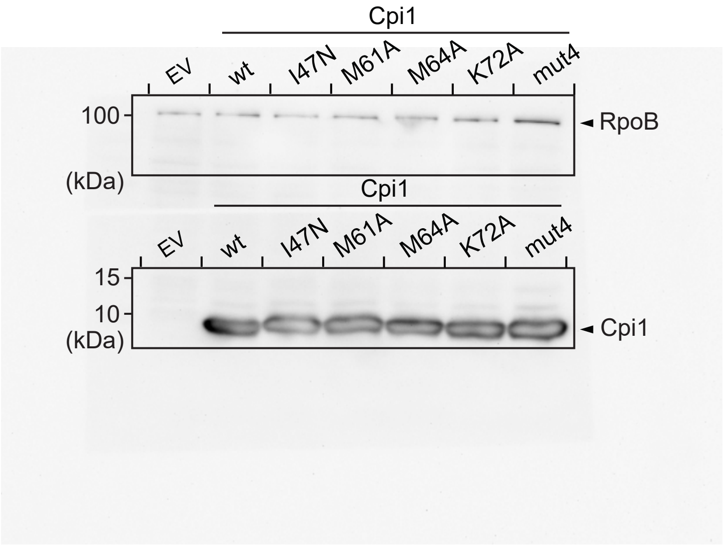

Supplementary Figure 5

Supplementary Figure 5A

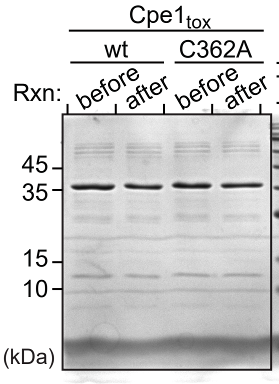

Supplementary Figure 5B

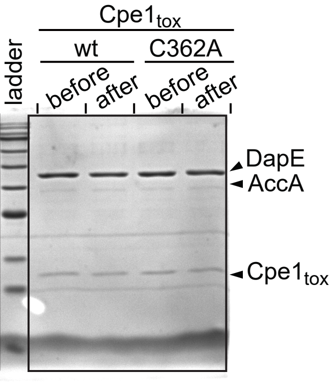

Supplementary Figure 5C

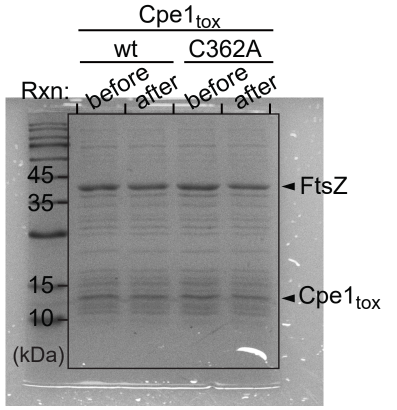

Supplementary Figure 5D

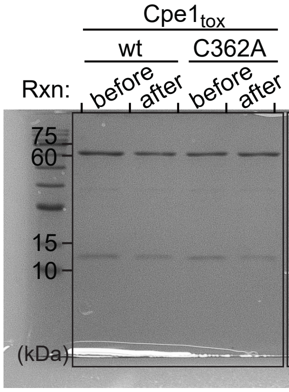

Supplementary Figure 5E

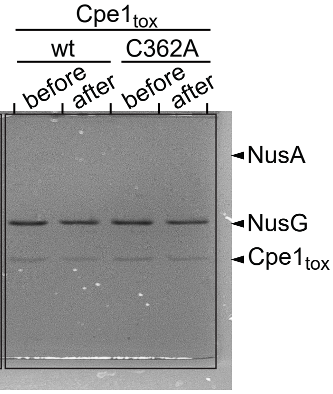

Supplementary Figure 5F

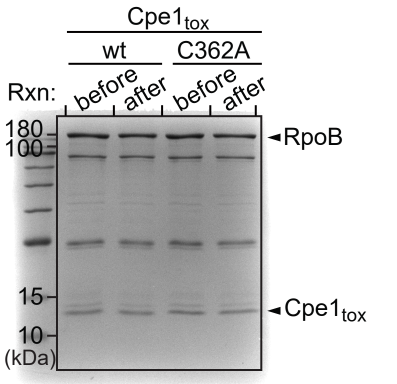

Supplementary Figure 5G

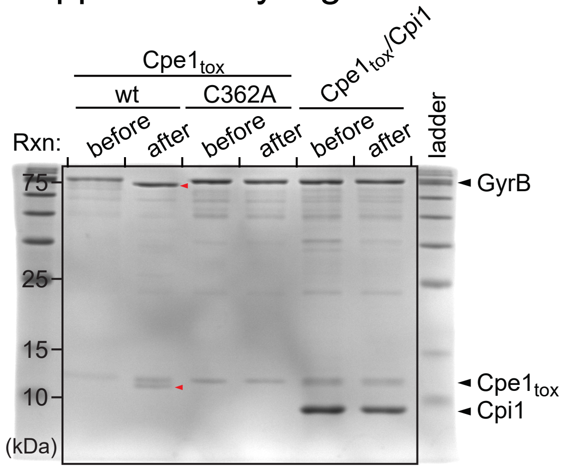

Supplementary Figure 5H

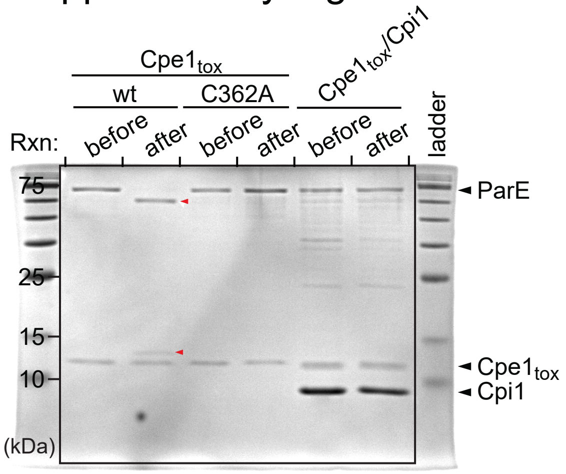

Supplementary Figure 9

Supplementary Figure 9D

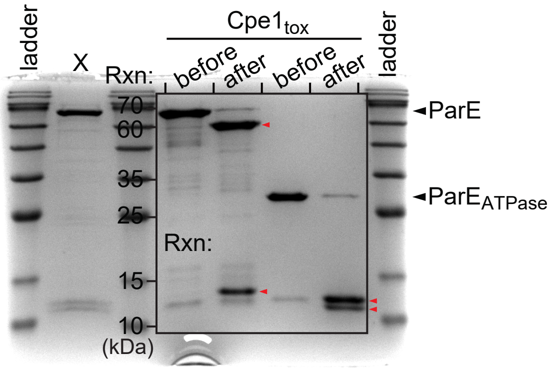

Supplementary Figure 10

Supplementary Figure 10A

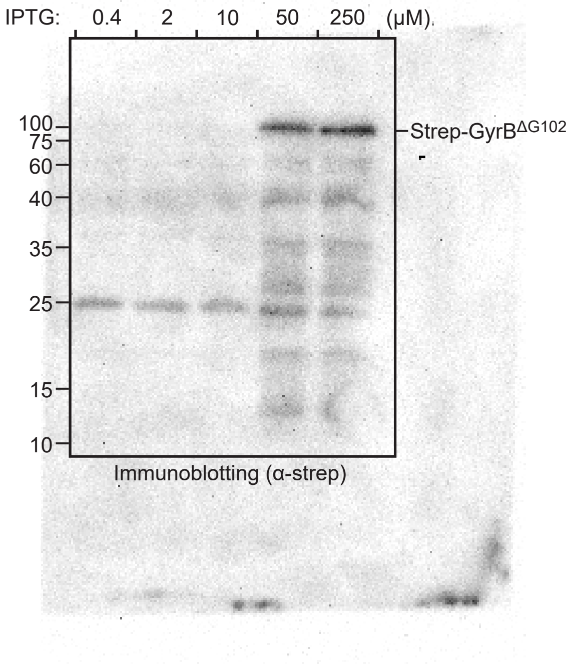

Supplementary Figure 10B

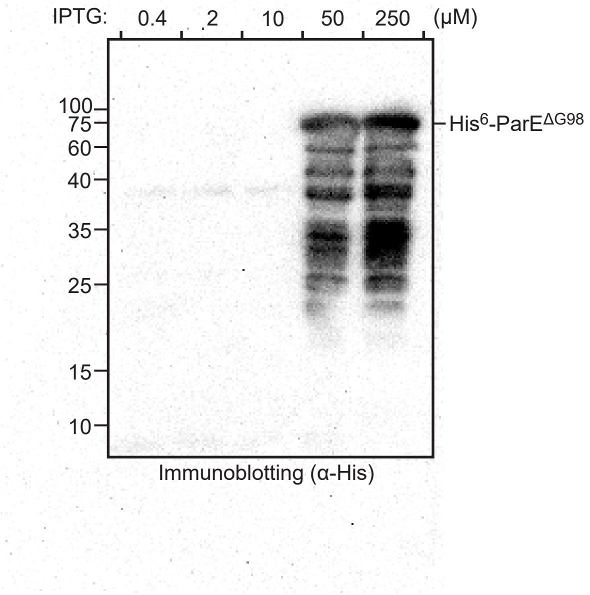

Supplement: S1 Raw Images — (PDF) [file pbio.3003208.s021.pdf]
