# Supplementary material for: An interbacterial cysteine protease toxin inhibits cell growth by targeting type II DNA topoisomerases GyrB and ParE
Source: PLoS Biol. 2025 May 27;23(5):e3003208. doi: 10.1371/journal.pbio.3003208 (PMC12136450; doi:10.1371/journal.pbio.3003208)
Supplement: Attachment — Submitted filename: 154672_1_rebuttal_3340614_stsjyh.pdf [file pbio.3003208.s022.pdf]

Full Revision

Manuscript number: RC-2025-02884
Corresponding author(s): See-Yeun, Ting

Point-by-point description of the revisions
Reviewer comments in **BLACK** and author responses in **BLUE**.

Reviewer #1 (Evidence, reproducibility, and clarity)
The manuscript by Song et al presents evidence to show that the predicted cysteine protease type 6 secretion system (T6SS) effector Cpe1 inhibits target cell growth by cleaving type II DNA Topoisomerases GyrB and ParE. The authors determined the structure of the protein complex formed by Cpe1 and its immunity protein Cpi1, which allowed them to reveal the mechanism of inhibition. Moreover, the authors identified type II DNA topoisomerases GyrB and ParE as the targets of Cpe1. Overall, the major conclusions were well supported by experimental data of high quality. The findings have expanded our appreciation of the mechanism utilized by T6SS effectors to inhibit target cell growth.

We thank the reviewer for their positive remarks and valuable suggestions to improve this manuscript.

Major comments
1. To better establish that GyrB and ParE are the sole targets of Cpe1, the authors should express the GG mutant in target cells and determine whether these cells become resistant to Cpe1-mediated killing (inhibition). They can also determine whether co-expression of the cleavage resistant mutants suppresses the toxicity of Cpe1.

We appreciate the reviewer's suggestion to investigate additional substrates of Cpe1 beyond GyrB and ParE, which may not have been fully captured in our crosslinking-mass spectrometry experiments due to technical limitations or low protein abundance. To address this topic, we generated target cells heterologously expressing cleavage-resistant GyrB and ParE variants ($GyrB^{\Delta G102}$ and $ParE^{\Delta G98}$) that are not susceptible to Cpe1, as described in our original manuscript (**Figures 3h, i**). We performed both Cpe1 expression assay and competition assay to assess if expression of the cleavage-resistant variants suppresses Cpe1 toxicity (**Author Response Figures 1a, b**). However, we did not observe a substantial protective effect. While this outcome could suggest that GyrB and ParE are not the sole targets of Cpe1, alternative explanations are also plausible. In the Cpe1 expression assay, high levels of Cpe1 could still act on endogenous wild-type GyrB and ParE, and although we attempted to increase variant expression, precise quantification remains challenging. In the competition assay, highly active Cpe1 may have continued to target wild-type substrates throughout the experiment, potentially masking any protective effect. Additionally, reduced activity of the mutant proteins could contribute to the observed results. Finally, deletion of the global repressor H-NS in the Cpe1-producing *E. coli* strain may have induced other interbacterial competition mechanisms[1], leading to growth inhibition independently of Cpe1. Addressing these questions comprehensively would require a more systematic investigation under a wider range of conditions. We consider this an important avenue for future studies.

Author Response Figure 1

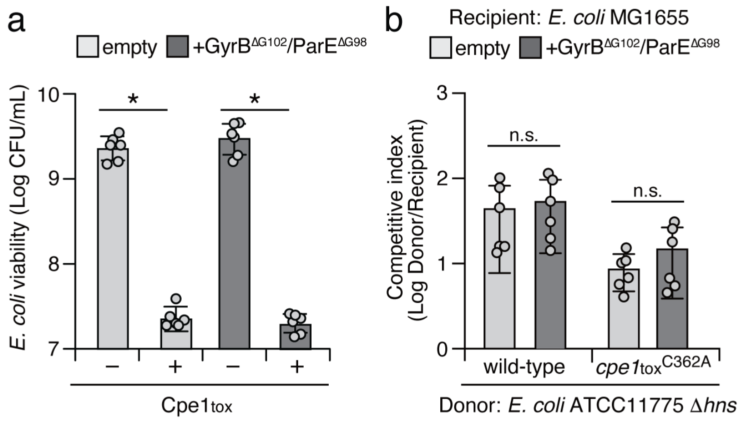

Author Response Figure 1. Assessing the substrate specificity of $Cpe1_{tox}$.
(**a**) Viable *E. coli* cells recovered from plating cultures carrying plasmids expressing the indicated proteins. *E. coli* co-expressing $Cpe1_{tox}$ with $GyrB^{\Delta G102}$ and $ParE^{\Delta G98}$ was incubated in media containing 0.5% arabinose and/or 0.25mM IPTG to induce protein expression and validate suppression of Cpe1 toxicity. (**b**) Bacterial competition experiments were conducted on solid agar over a 4-hour period between the indicated donor and recipient strains. *E. coli* ATCC-11775 Δ*hns* served as the donor strain, whereas *E. coli* MG1655 carrying plasmids encoding pBAD-regulated $GyrB^{\Delta G102}$ and $ParE^{\Delta G98}$ was used as the recipient strain. Culture of the recipient cells, as well as the competition assay, was performed in the presence of 0.5% arabinose for expression of the cleavage-resistant mutants. The data in **(a)** and **(b)** are shown as the mean ± SD; $n = 6$. Both datasets are representative experiments from at least three independent experiments. Asterisks indicate statistically significant differences ($p < 0.05$) in viability between populations **(a)** or competitive indices of a given donor strain toward the indicated recipients **(b)**.

2. Results in Figure 7 clearly show that Cpi1 is capable of displacing ParE from Cpe1 due to higher affinity. Yet, the "competitive inhibition model" described in the last result section does not completely match what is really happening in Cpe1-mediated interbacterial competition. If Cpi1 is in the target cell, it would more likely engage the incoming Cpe1 before it can interact with ParE or GyrB, so competition does not occur in this scenario. Similarly, in the predatory cells expressing Cpe1 and Cpi1, these two proteins will form a stably protein complex, and no competition with the target will occur. The authors should reconsider their model.

We thank the reviewer for their comments and appreciate the opportunity to clarify this point. First, we believe the reviewer is referring to **Figure 5** rather than **Figure 7**. In our model, the primary role of immunity proteins in interbacterial competition is to neutralize cognate toxins and prevent self- or kin-intoxication. These immunity proteins exhibit high specificity and strong binding affinity toward their associated toxins, ensuring effective protection[2]. In predatory cells, immunity proteins are typically co-expressed with their corresponding toxins,

likely enabling immediate suppression upon translation. During kin competition, immunity proteins can protect cells even after foreign toxins engage their substrates.

Our results demonstrate that Cpi1 binds Cpe1 with higher affinity than its substrates and can displace them from pre-formed Cpe1-substrate complexes (**Figures 5b-f**). This aligns with the established function of immunity proteins in interbacterial competition and provides a mechanistic basis for how they confer protection, even when toxins have initially engaged their targets[2]. We acknowledge the reviewer's point that in both scenarios—whether in the recipient cell or the toxin-producing cell—Cpe1 may first encounter Cpi1. However, our model underscores that Cpi1 not only binds at the substrate site but also exhibits superior affinity for Cpe1, ensuring robust protection against Cpe1-mediated toxicity.

Minor comments

1. "Intoxication" was used throughout the text numerous times to describe the activity of Cpe1. Looking in the Marriam-Webster dictionary, "Intoxication" means "a condition of being drunk". This word should be replaced with "toxicity" or some other terms in this line.

We thank the reviewer for this comment. We acknowledge that the term "intoxication" is commonly associated with alcohol consumption, yet the Merriam-Webster dictionary also defines it as "an abnormal state that is essentially a poisoning" (https://www.merriam-webster.com/dictionary/intoxication). This definition aligns with its well-established usage in the field of interbacterial competition to describe the effects of interbacterial toxins during antagonism[3-5], which we have adopted in our manuscript. However, we appreciate the reviewer's concern and remain open to revising the terminology if deemed necessary for clarity.

2. Lines 46-48, references on contact-dependent killings by these systems mentioned should cited. Ref. 9 cited does NOT cover the information at all.

We thank the reviewer for this comment. We have revised the citation and now reference studies that specifically describe contact-dependent killing systems in the relevant sentences (**Lines 45–50**).

3. "characterizations" should be "characterization".

We have now modified the sentence as requested (**Line 69**).

4. Line 229 "Cpe1-Bpa monomers" should be " apo Cpe1-Bpa". The results cannot distinguish whether these bands are monomers or multimers.

We appreciate the reviewer's careful assessment of our manuscript. The results in **Line 233** (**Figure 3c**) show the enrichment of His-tagged proteins, including crosslinked complexes and overproduced Cpe1-Bpa. Based on the molecular weight marker, the Cpe1-Bpa bands appear between 10–15 kDa, consistent with the molecular weight of Cpe1 monomers (**Figure 3a**). Therefore, we have labeled this band as "Cpe1-Bpa monomers" and maintained

this terminology throughout the text. This designation aligns with previous studies utilizing site-specific crosslinking via Bpa incorporation[6,7].

5. Line 283, was the mutation deletion? Substitution was used I think.

We thank the reviewer for highlighting this point. The GyrB and ParE mutants used to confirm the cleavage sites were deletion mutants, with a single glycine removed from the predicted double-glycine motifs. We have now revised the text for clarity (**Lines 285–290**).

6. Lines 439-444 the discussion should be extended to include other bacterial toxins that target type II DNA topoisomerases (e.g. PMID: 26299961 and PMID: 26814232).

We appreciate the reviewer's suggestion. The studies referenced (PMID: 26299961 and PMID: 26814232) describe FicT toxin with adenylyl transferase activity that target and post-translationally modify GyrB and ParE at their ATPase domains, highlighting a potential hotspot for topoisomerase inhibition. We have now incorporated an additional paragraph in the Discussion section to describe these findings (**Lines 424–439**).

Reviewer #1 (Significance)

The authors determined the structure of the protein complex formed by Cpe1 and its immunity protein Cpi1, which allowed them to reveal the mechanism of inhibition. Moreover, the authors identified type II DNA topoisomerases GyrB and ParE as the targets of Cpe1. Overall, the major conclusions were well supported by experimental data of high quality. The findings have expanded our appreciation of the mechanism utilized by T6SS effectors to inhibit target cell growth.

We sincerely thank the reviewer for their positive comments and for the suggestions to improve our manuscript.

Reviewer #2 (Evidence, reproducibility, and clarity)

The manuscript, titled "An Interbacterial Cysteine Protease Toxin Inhibits Cell Growth by Targeting Type II DNA Topoisomerases GyrB and ParE", describes how an effector family was identified and characterized as a papain-like cysteine protease (PLCP) that negatively impacts bacterial growth in the absence of its co-encoded immunity protein. This thorough report includes (1) bioinformatic analysis of prevalence, finding this PLCP effector encoded in many gram-negative bacteria, (2) confirming conservation of catalytic active site via structural (crystallographic) analysis, as well as visualizing contacts with the immunity protein, (3) validation of results using growth studies combined with mutagenesis, (4) using a cell-based cross-linking method to pull out potential targets, which were subsequently identified via mass spectrometry, (5) validation of these results using in vitro protease assays with purified (potential) substrates, including verification of the motif recognized on the substrate(s), and cell-based phenotype analyses, and finally, (6) demonstrating competition between immunity protein and ParE substrate using an in vitro pull-down approach. Overall, this is a strong body of work with compelling conclusions that are well supported by multiple experimental approaches.

We appreciate the reviewer for their positive comments regarding our original submission.

Major comments

The claims made based on the presented results are well supported, including that this PLCP effector toxin is widespread, is neutralized in a competitive mechanism by its immunity partner, and that it effectively cleaves both GyrB and ParE (subunits of bacterial type II topoisomerases) at a conserved motif, resulting in suppression of bacterial cell growth via mis-regulating chromosome segregation. No additional experiments are needed to further validate these results, and the authors are commended on the cell-based and in vitro studies to deduce very specific mechanisms and structural details.

We appreciate the reviewer's positive feedback.

Minor comments

While the writing and data presentation are extremely clear, in general I recommend the authors indicate the level(s) of replication for experiments. Figure legends generally note that mean values with standard deviations are shown, but I did not find where the number of replicates (and independent versus technical) were listed.

We appreciate the reviewer's suggestion. We have now revised the manuscript to specify the levels of replication (independent vs. technical) for each experiment in the figure legends, particularly in **Figures 2 and 3**.

The figures are very clear, but in many instances the addition of PLCP toxin is indicated as "before" and "after"; while a modest change, I recommend altering this to some type of "-" and "+" type nomenclature rather than a time-based notation (especially as presumably both samples were treated identically, just with or without protease).

We thank the reviewer for this helpful comment. In **Figures 3 and Supplementary Figures 5, 9**, we used "before" and "after" to indicate the time points for *in vitro* cleavage assays verifying Cpe1 cleavage. To minimize variations

between reactions, the catalytic mutant $Cpe1_{tox}$ ($Cpe1_{tox}^{C362A}$) was used as a comparison rather than a reaction without $Cpe1_{tox}$. In these assays, duplicate reaction mixtures were prepared: one was denatured immediately after preparation ("before" reaction) to serve as a baseline, while the other was incubated to allow enzymatic activity ("after" reaction). This labeling clarifies the comparison between initial and processed samples. We believe this approach clearly distinguishes the effects of Cpe1 activity and provides a reliable basis for assessing proteolysis in our assays.

I also suggest quantifying the intensities of the gel images presented in Figure 5c, d (for example, Cpe1 intensity as a ratio to that of the ParE ATPase domain), to make the interpretation even more evident.

We thank the reviewer for the valuable suggestion to quantify the signal intensities of the gel images presented in **Figures 5c, d**. We have now included the quantification results in **Supplementary Figures 9e, f** and have updated the respective text in the manuscript (**Lines 826-828 and 1066-1087**).

Crystallographic structure: the PDB report notes some higher-than-expected RZR (RSRZ) scores; I interpret this to mean that there was strain around the catalytic site of one of the two toxins in the asymmetric unit, or that this copy was less well ordered. The RZR outliers likely arise from non-optimal weighting for geometric restraints. While no figures of electron density are presented, these modest outliers are not expected to alter the conclusions reached in the current work. One point of interest that is not addressed, however, is if any variance between the two complexes in the asymmetric unit are noted? A passage compares the current toxins to others in the larger subfamily and notes a rotation of a side chain is needed to superpose (Line 159). Can the authors please clarify around which bond this rotation is needed, and if both copies in the asymmetric unit are in the same orientation at this site?

We appreciate the reviewer's insightful comments.

1. We have provided the electron density map for the RSR-Z outlier residues along with the model (**Author response Figure 2a**). These outlier residues are located at the loop regions of a molecule within the asymmetric unit in the crystal (Chain B). As a result, the electron density for their side chains appears to be noisier compared to residues in the well-folded regions, leading to higher RSR-Z scores. Notably, when we superimposed the models of two complexes within the asymmetric unit, the calculated RMSD value was 0.402 Å (**Author response Figure 2b**), indicating that the two models are structurally very similar and that these residues are properly assigned. Therefore, the RSR-Z outliers do not significantly impact the overall structure.

2. Here, we provide a zoomed-in view of **Figure 2d**, highlighting the superimposed crystal structures of Cpe1 and the closely related PLCPs, ComA and LahT (**Author response Figure 2c**). As shown, the side chain of the catalytic cysteine residue in ComA adopts a different orientation, positioning it slightly farther from the homologous residues in Cpe1 and LahT. However, since the backbone and catalytic pockets remain structurally intact, we believe that this deviation arises due to results from crystal packing effects rather than an inherent functional distinction. We have now modified the main text (**Lines 159-166**) to clarify this and prevent any potential misinterpretation.

Author Response Figure 2

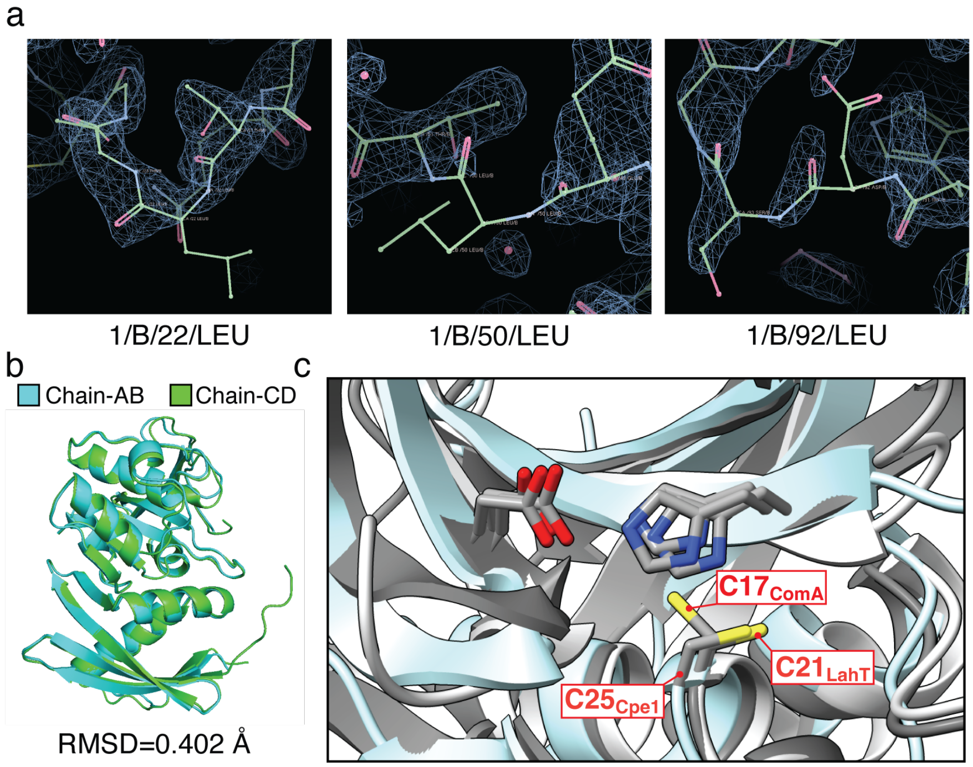

Author Response Figure 2. Analysis of the Cpe1-Cpi1 crystal structure model.
(**a**) The electron density map for the RSR-Z outlier residues on the B-chain of the model. (**b**) Superimposition of two Cpe1-Cpi1 complexes in the asymmetric unit (Chain-AB and Chain-CD) and the RMSD of the structural alignment. (**c**) Zoomed-in view of the catalytic pocket of superimposed $Cpe1_{tox}$, $ComA_{pep}$, and $LahT_{pep}$ with their respective catalytic cysteines labeled.

<u>Reviewer #2 (Significance)</u>

Bacteria encode numerous effectors to successfully compete in natural environments or to mediate virulence; these effectors are typically associated with type VI secretion system machinery or referred to as contact dependent inhibition systems. The current work has identified a sub-family of papain-like cysteine protease effectors that are unique by targeting type II topoisomerases. Among the actionable findings is the identification of both the specific site of interaction with the topo substrates, as well as the specific motif recognized for cleavage. This should enable the field to move forward probing for this activity with other toxins and substrates. The insights provided by the competitive neutralization mechanism also stand out as an important contribution that can be more broadly applied. Within the literature, few effector targets are identified, making the current study stand out as impactful by the well-executed experiments that directly support the conclusions.

While the current study has strong elements of novelty and is complete, it also nicely sets up future studies for remaining open questions. For example, does the nucleotide-bound status of the ATPase domain, or other catalytic intermediate, impact the susceptibility of topoisomerases to cleavage? Is this identified motif found in other ATPase domains? Is the negative supercoiling activity unique to gyrase also impacted, or is the phenotypic mechanism of cell toxicity reliant only on chromosome segregation? What types of kinetic parameters do this class of toxins demonstrate, and does sequence variability alter this? These ideas are a testament to the intriguing study as presented, capturing the readers' curiosity for additional details that are clearly beyond the scope of the current work.

I anticipate this work will be of interest to the broad field of microbiologists that study interbacterial communication as well as pathogenic mechanisms. While the research is largely fundamental in nature, it is wide in scope with applications to many gram-negative bacteria that inhabit a myriad of niches. The work will also be of interest to specialists in topoisomerases, as the list of toxins that target these essential enzymes is growing and the therapeutic utility of topoisomerase inhibition remains vital. My interest lies in the latter, in toxin-mediated inhibition of topoisomerase enzymes as a means to alter bacterial cell growth. While I have strong expertise in structural biology, I am lacking in expertise for mass spectrometry. I note this because this method was used for the identification of the target substrate.

We appreciate the reviewer's insightful discussion and interest in our study. We agree that further investigations are crucial to address the open questions posed, and we have initiated work on some of these avenues.

For example, considering Cpe1's specificity for the ATPase domain of GyrB and ParE, we have begun examining whether Cpe1 targets other ATPase domains by searching for the consensus sequence or double glycine motifs in the sequences of ATPase domains beyond GyrB and ParE. Among the 42 *E. coli* ATPase domains identified by the PEC database[8], we found several with double glycine residues. However, none contained the exact LHAGGKF consensus sequence identified in GyrB and ParE, which are targeted by Cpe1 (**Author Response Figure 3**). These findings suggest that Cpe1 is less likely to target other ATPase domains. Nonetheless, due to Cpe1's potential tolerance of certain variations within the consensus sequence, we cannot draw a definitive conclusion without further investigation into the cleavage sites.

Another critical open question is the impact of Cpe1-mediated cleavage on the function of GyrB and ParE. To address this topic, we have begun investigating if Cpe1 cleavage affects the ATPase activity of these proteins. As expected, our biochemical analysis has demonstrated a significant decrease in ATP hydrolysis in the presence of active $Cpe1_{tox}$, but not in the presence of the catalytic mutant $Cpe1_{tox}^{C362A}$ (**Author response Figures 4a, b**). These results confirm that the ATP-dependent activities of both GyrB and ParE are disrupted following Cpe1 cleavage[9]. Previous work on FicT toxin that inhibits GyrB and ParE ATPase activity through post-translational modification found that ATP-dependent activities such as DNA supercoiling, relaxation, and decatenation were inhibited[10,11]. Interestingly, GyrB's relaxation of negative supercoiled DNA, which does not require ATP, was also affected to some extent. This outcome raises the question as to whether Cpe1-cleaved GyrB results in similar downstream defects. Investigating this possibility would provide valuable insights into Cpe1's mode of action,

although we feel doing so is beyond the scope of the current study. Consequently, we view this as an important area for future research.

Finally, regarding the potential applications of Cpe1, we are interested in further investigating its enzymatic specificity and properties. In this study, we analyzed the binding kinetics between Cpe1 and its substrate (**Figure 5f**) and currently we are endeavoring to characterize the kinetics of Cpe1-mediated proteolysis. To better probe hydrolytic dynamics, we plan to utilize a substrate with a reporting group (such as a chromogenic or fluorogenic leaving group) to monitor cleavage over time. We could achieve this by designing a recombinant substrate based on our knowledge of Cpe1's native substrates (GyrB and ParE) and the target sequence ("LHAGGKF"). Alternatively, a secondary reaction leading to colorimetric changes could be employed for detection. We consider this an exciting research direction and an important next step for this study.

Overall, we are grateful for the reviewer's recognition of the novelty and importance of our work in advancing the understanding of interbacterial toxins and their inhibitory effects on topoisomerases. We plan to further investigate the consequences of Cpe1 cleavage on GyrB and ParE and to explore Cpe1 kinetics and its mechanistic actions in more detail. This will not only deepen our understanding of bacterial toxin-mediated inhibition but may also provide critical insights into strategies for targeting type II DNA topoisomerases. The reviewer's insightful feedback has proven invaluable in shaping our ongoing and future research directions.

Author Response Figure 3

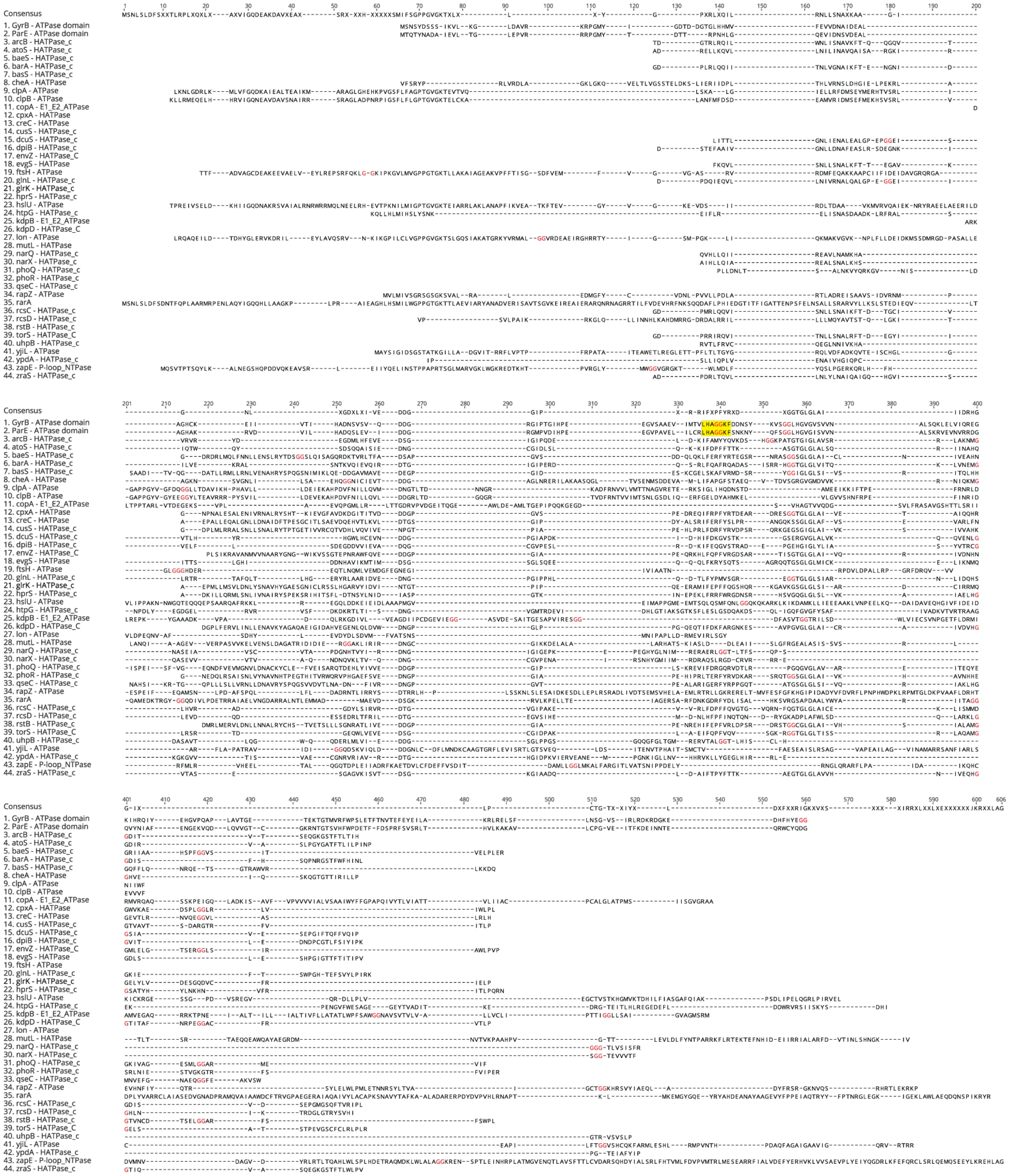

Author Response Figure 3. Sequence alignment of *E. coli* ATPase domains.
Forty-two ATPase domains from *E. coli* were extracted and aligned with those of GyrB and ParE. The GG motifs are shown as red letters. The Cpe1 cleavage site, LHAGGKF, is highlighted by a yellow background.

Author Response Figure 4

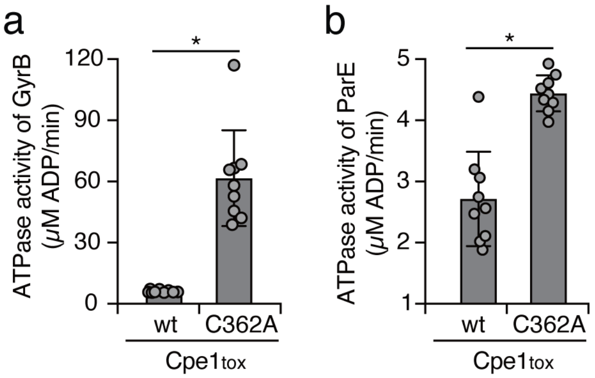

Author Response Figure 4.
(**a, b**) ATPase activity of GyrB (**a**) and ParE (**b**) in the presence of wild-type or catalytic mutant $Cpe1_{tox}$. The data in (**a**) and (**b**) are shown as the mean ± SD of three independent experiments ($n = 3$). Asterisks indicate statistically significant differences ($p < 0.05$) in enzyme activity.

Reviewer #3 (Evidence, reproducibility, and clarity)
Bacterial warfare in microbial communities has become illuminated by recent discoveries on molecular weapons that allow contact-dependent injection of bacterial toxins between competitors. Among the best characterized systems are the type VI secretion system (T6SS) or the contact-dependent inhibition (CDI) system (i.e. some of the T5SSs). These systems are delivering a plethora of toxins with various biochemical activities and a broad range of targets. In recent years many such toxins have been characterized and their relevance in pointing at appropriate drug targets is increasing.

In this study the authors built on a previously published association of a family of proteins, papain-like cysteine proteases (PLCPs), with their delivery by T6SS or CDI into target bacterial cells. Whereas this observation is not particularly novel, the findings that this set of proteins, that the authors called now Cpe1, can specifically target bacterial proteins such as ParE and GyrB, so that it affects chromosome partitioning and cell division, is groundbreaking. The authors are clearly demonstrating that Cpe1 cleaves their target proteins at double glycine recognition site which is in line with previous characterization of such proteases when fused to a particular category of ABC transporters. Even more remarkably they can show using biochemical approaches that Cpi1 is a cognate immunity for CpeI, preventing its activity, not by interfering with the catalytic site, but instead with the substrate binding site. The mechanism of competitive inhibition between immunity and substrate is also substantiated by biochemical data.

We sincerely appreciate the reviewer's interest in and support of our study.

Major comments
- This is a very well conducted study which combines bacterial genetics and phenotypes with excellent biochemical evidence.

We thank the reviewer for their positive comments.

- There are 8 targets identified for Cpe1 and yet only two are cleaved by the enzyme. It is intriguing that FtsZ is one identified target by the pull down but not confirmed for cleavage. The authors rules this as false positive but the cell division defect associated with Cpe1 activity would be consistent here. Are there any double glycine in FtsZ that could be identified as cleavage site? Is it possible that slightly different incubation conditions may promote degradation of FtsZ?

We appreciate the reviewer's thoughtful comment regarding FtsZ as a potential substrate of Cpe1. This was indeed an intriguing possibility, especially given the cell division defects observed following Cpe1 intoxication. Early on in the project, we also identified FtsZ as a Cpe1 interactor in our proteomic crosslinking assays, which further fueled the hypothesis that FtsZ might be a target.

To explore this possibility, first we examined the FtsZ protein sequence for potential Cpe1 cleavage sites and identified several double glycine motifs (**Author response Figure 5a**). However, none of these motifs matched the consensus sequence identified in GyrB and ParE, which is LHAGGKF, a sequence that we have shown to be critical for Cpe1 cleavage activity. In an effort to better understand if FtsZ could still be cleaved by Cpe1, we conducted additional cleavage assays under various conditions (**Author response Figure 5b**). We tested different incubation temperatures, including increasing the temperature to 37 °C, and extended the reaction time to overnight. However, we did not observe any cleavage of FtsZ under these conditions. Given that FtsZ undergoes significant conformational changes upon binding to GTP[12], we also considered the possibility that the GTP-bound form of FtsZ might be cleaved by Cpe1. However, even under those conditions, no significant cleavage of FtsZ was detected (**Author response Figure 5b**). Based on these results, we do not have any evidence to support that FtsZ is a target of Cpe1. The observed cell division defects are more likely a secondary effect resulting from the cleavage of GyrB and ParE, direct targets of Cpe1 that are crucial for chromosome segregation.

- Could it be structurally predicted whether the GG of ParE or GyrB is fitted into the catalytic site of Cpe1.

We appreciate the reviewer's insightful question regarding the structural prediction of the GG motif of ParE and GyrB fitting into the catalytic site of Cpe1. To address this possibility, we used Alphafold 3 to predict the interaction structure between Cpe1 and its substrates[13]. The resulting model of Cpe1 interacting with the ATPase domain of GyrB ($GyrB_{ATPase}$) is shown in **Supplementary Figure 9c**. As illustrated, the loop of the GyrB ATPase domain containing the consensus targeting sequence ("LHAGGKF") fits into the catalytic site of Cpe1, with the GG motif positioned closest to the catalytic cysteine residue, which likely facilitates hydrolysis. We also attempted to model the interaction between Cpe1 and the ATPase domain of ParE. However, confidence for this model was lower (ipTM = 0.74, pTM = 0.71), possibly due to Alphafold's preference for certain protein configurations. To gain a more accurate understanding of how Cpe1 binds and recognizes its substrates, we are currently working on co-crystallizing $Cpe1_{tox}$ with GyrB and ParE. This long-term project aims to provide precise structural insights into the Cpe1-substrate interaction and further elucidate the mechanism of cleavage.

Author Response Figure 5

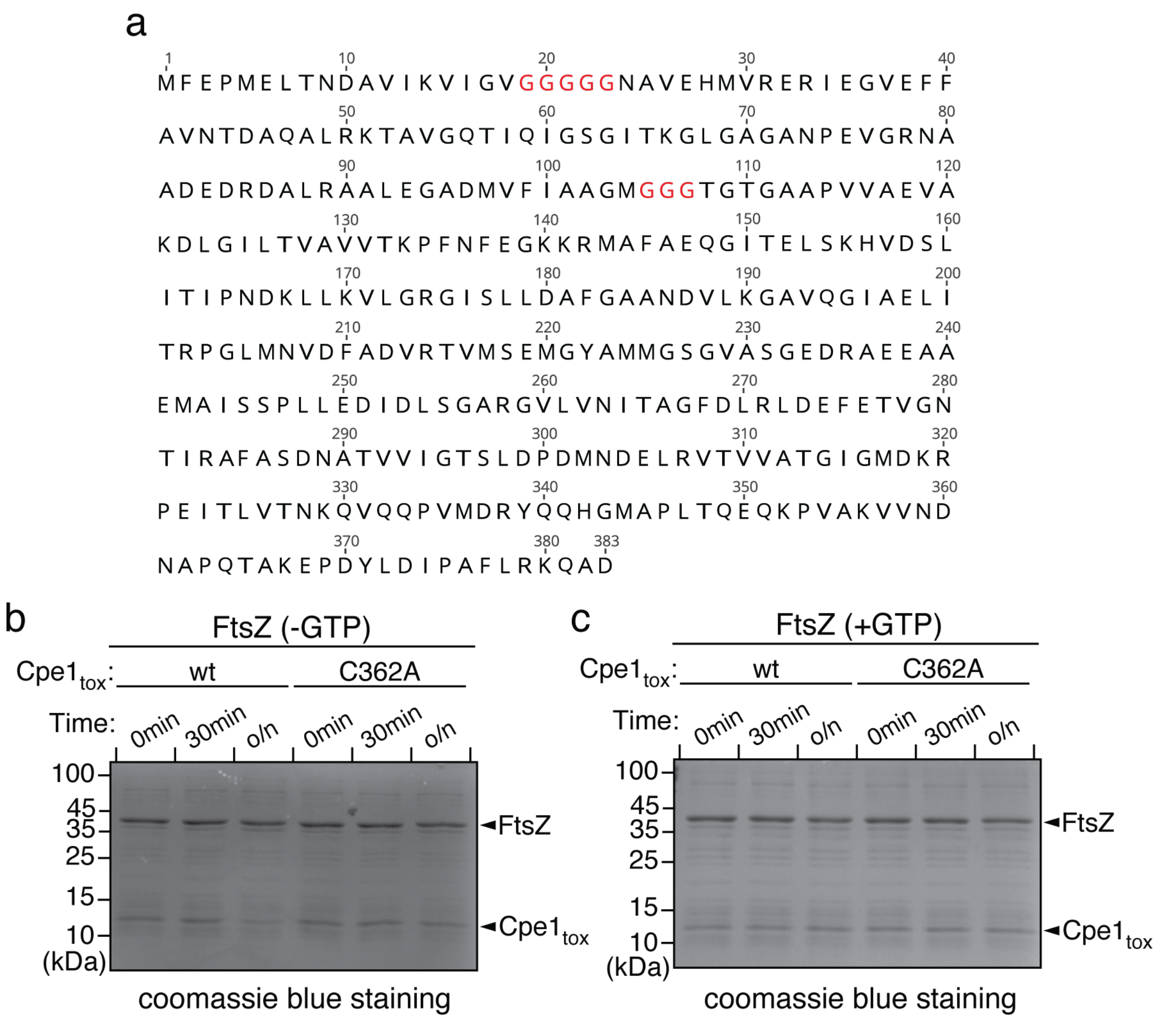

Author Response Figure 5. Analysis of FtsZ as a putative Cpe1 substrate.
(**a**) The protein sequence of FtsZ. The GG motifs are shown in red letters. (**b, c**) *In vitro* cleavage assays of FtsZ. Purified FtsZ was incubated with purified Cpe1$_{tox}$ (wild-type or catalytic mutant) at 37 °C with (**b**) or without (**c**) the presence of 5 mM GTP and 10 mM $MgCl_2$ for different timeframes to assess hydrolysis by Cpe1$_{tox}$.

<u>Minor comments</u>

- The authors described a family of proteases, PLPCs, and characterized one here called Cpe1. Not clear whether this is a generic name or one specific protein from one particular bacterial species. Indeed, it is unclear from which bacterial strain the Cpe1 protein studied here originates.

We thank the reviewer for this comment and apologize for the lack of clarity. To provide better context, we have now revised the manuscript (**Lines 136-137 and 141-145**) to clearly state that the Cpe1 protein characterized in this study originates from *E. coli* strain ATCC 11775.

- It may be worth to emphasize that the Cpe1 domain is found in all possible configurations as T6SS cargo and that is to be linked to VgrG, PAAR or Rhs.

Thank you for this suggestion. We have revised the manuscript accordingly to emphasize this point (**Lines 106-109**).

- Line 49 the authors could indicate that the Esx system is also known as type VII secretion system (T7SS).

Thank you for this suggestion. We have revised the manuscript accordingly (**Line 48-50**).

- Line 113 it may be better to use Proteobacteria instead of Pseudomonadota

We have revised the manuscript (**Lines 114-115**) as suggested by the reviewer. It is important to note that following the recent decision by the International Committee on Systematics of Prokaryotes (ICSP) to amend the International Code of Nomenclature of Prokaryotes (ICNP) and formally recognize "phylum" under official nomenclature rules[14,15], the taxonomy database used in our analysis has adopted the updated nomenclature. To ensure consistency, we followed this updated nomenclature throughout the original manuscript.

Reviewer #3 (Significance)

This is an excellent piece of work. The characterization of Cpe1 might look poorly novel at the start when compared to previous studies. Yet the findings go crescendo by characterizing original mechanisms of action of the cognate immunity, and by identifying the molecular target of Cpe1. This is providing real conceptual advance in the T6SS field and not just reporting yet another T6SS toxin.

As a T6SS expert I genuinely feel that these findings are groundbreaking and could be targeted to broad audience since the possible implications of these observations for future antimicrobial drugs discovery or therapeutic approaches is highly relevant.

We sincerely appreciate the reviewer’s positive remarks and support of our study.

References

1. Ishihama, A., and Shimada, T. (2021). Hierarchy of transcription factor network in Escherichia coli K-12: H-NS-mediated silencing and Anti-silencing by global regulators. FEMS Microbiol Rev *45*. 10.1093/femsre/fuab032.
2. Hersch, S.J., Manera, K., and Dong, T.G. (2020). Defending against the Type Six Secretion System: beyond Immunity Genes. Cell Rep *33*, 108259. 10.1016/j.celrep.2020.108259.
3. Russell, A.B., Singh, P., Brittnacher, M., Bui, N.K., Hood, R.D., Carl, M.A., Agnello, D.M., Schwarz, S., Goodlett, D.R., Vollmer, W., and Mougous, J.D. (2012). A widespread bacterial type VI secretion effector superfamily identified using a heuristic approach. Cell Host Microbe *11*, 538-549. 10.1016/j.chom.2012.04.007.
4. Jana, B., Fridman, C.M., Bosis, E., and Salomon, D. (2019). A modular effector with a DNase domain and a marker for T6SS substrates. Nat Commun *10*, 3595. 10.1038/s41467-019-11546-6.
5. Halvorsen, T.M., Schroeder, K.A., Jones, A.M., Hammarlof, D., Low, D.A., Koskiniemi, S., and Hayes, C.S. (2024). Contact-dependent growth inhibition (CDI) systems deploy a large family of polymorphic ionophoric toxins for inter-bacterial competition. PLoS Genet *20*, e1011494. 10.1371/journal.pgen.1011494.

6. Nguyen, T.T., Sabat, G., and Sussman, M.R. (2018). In vivo cross-linking supports a head-to-tail mechanism for regulation of the plant plasma membrane P-type H(+)-ATPase. J Biol Chem *293*, 17095-17106. 10.1074/jbc.RA118.003528.
7. Liu, Y., Yu, J., Wang, M., Zeng, Q., Fu, X., and Chang, Z. (2021). A high-throughput genetically directed protein crosslinking analysis reveals the physiological relevance of the ATP synthase 'inserted' state. FEBS J *288*, 2989-3009. 10.1111/febs.15616.
8. Yamazaki, Y., Niki, H., and Kato, J. (2008). Profiling of Escherichia coli Chromosome database. Methods Mol Biol *416*, 385-389. 10.1007/978-1-59745-321-9_26.
9. Reece, R.J., and Maxwell, A. (1991). DNA gyrase: structure and function. Crit Rev Biochem Mol Biol *26*, 335-375. 10.3109/10409239109114072.
10. Harms, A., Stanger, F.V., Scheu, P.D., de Jong, I.G., Goepfert, A., Glatter, T., Gerdes, K., Schirmer, T., and Dehio, C. (2015). Adenylylation of Gyrase and Topo IV by FicT Toxins Disrupts Bacterial DNA Topology. Cell Rep *12*, 1497-1507. 10.1016/j.celrep.2015.07.056.
11. Lu, C., Nakayasu, E.S., Zhang, L.Q., and Luo, Z.Q. (2016). Identification of Fic-1 as an enzyme that inhibits bacterial DNA replication by AMPylating GyrB, promoting filament formation. Sci Signal *9*, ra11. 10.1126/scisignal.aad0446.
12. Matsui, T., Han, X., Yu, J., Yao, M., and Tanaka, I. (2014). Structural change in FtsZ Induced by intermolecular interactions between bound GTP and the T7 loop. J Biol Chem *289*, 3501-3509. 10.1074/jbc.M113.514901.
13. Abramson, J., Adler, J., Dunger, J., Evans, R., Green, T., Pritzel, A., Ronneberger, O., Willmore, L., Ballard, A.J., Bambrick, J., et al. (2024). Accurate structure prediction of biomolecular interactions with AlphaFold 3. Nature *630*, 493-500. 10.1038/s41586-024-07487-w.
14. Oren, A., Arahal, D.R., Rossello-Mora, R., Sutcliffe, I.C., and Moore, E.R.B. (2021). Emendation of Rules 5b, 8, 15 and 22 of the International Code of Nomenclature of Prokaryotes to include the rank of phylum. Int J Syst Evol Microbiol *71*. 10.1099/ijsem.0.004851.
15. Oren, A., and Garrity, G.M. (2021). Valid publication of the names of forty-two phyla of prokaryotes. Int J Syst Evol Microbiol *71*. 10.1099/ijsem.0.005056.
