# Supplementary material for: An interbacterial cysteine protease toxin inhibits cell growth by targeting type II DNA topoisomerases GyrB and ParE
Source: PLoS Biol. 2025 May 27;23(5):e3003208. doi: 10.1371/journal.pbio.3003208 (PMC12136450; doi:10.1371/journal.pbio.3003208)
Supplement: Attachment — Submitted filename: Song et al 2025 Response to Reviewers.pdf [file pbio.3003208.s024.pdf]

Manuscript number: PBIOLOGY-D-25-01040R1
Corresponding author(s): See-Yeun, Ting

Point-by-point description of the revisions
Reviewer comments in **BLACK** and author responses in **BLUE**.

Reviewer #1:

The results that overexpression of cleavage-resistant mutants of GyrB and ParE cannot confer resistance to Cpe1 in both co-expression and killing assays is unexpected, particularly when these mutants were expressed from multi-copy plasmids. The authors' explanation was not satisfactory. Did the authors examine the expression of these mutants by methods like immunoblotting? If these mutants were properly expressed in the testing strains, and these strains are still resistant to Cpe1-meidated killing, the results should be included in the paper and appropriately discussed to acknowledge that Cpe1 likely targets other essential proteins.

We thank the reviewer for this comment. We have performed additional immunoblotting experiments to directly assess the expression of the cleavage-resistant $GyrB^{\Delta G102}$ and $ParE^{\Delta G98}$ variants in target cells (***new* Supplementary Figure 10**). As shown in the Supplementary Figure, both proteins are robustly expressed, confirming that the lack of protection against Cpe1-mediated intoxication is not due to insufficient protein expression. These data suggest that the inability of the cleavage-resistant variants to confer protection cannot be solely attributed to expression levels and support the possibility raised by the reviewer that Cpe1 may act on additional essential targets. As described in the ***revised* Discussion (Lines 424-442)**, while Cpe1 specifically recognizes a conserved consensus motif (LHAGGKF) located in a loop region of both GyrB and ParE, an exhaustive bioinformatic search did not identify other *E. coli* proteins containing this exact sequence. However, we acknowledge that this does not preclude the possibility of additional substrates with partial sequence similarity or structural features compatible with Cpe1 recognition. To address this, we are actively investigating the contribution of individual residues within the consensus motif to determine the sequence and structural constraints that govern Cpe1 specificity.

We have also incorporated additional discussion to present alternative, non-mutually exclusive explanations for our observations. For instance, in the co-expression assay, it remains possible that high levels of Cpe1 continue to act on the endogenous wild-type GyrB and ParE despite elevated expression of the mutant forms. In the interbacterial competition assay, persistent targeting of wild-type proteins during the course of the competition experiment could similarly mask the protective effect of the mutants. Furthermore, we cannot exclude the possibility that the cleavage-resistant variants are partially compromised in activity. Lastly, we note that the Cpe1-producing strain lacks the global transcriptional repressor H-NS, which may induce additional antagonistic pathways that contribute to growth inhibition independently of Cpe1.

We now discuss these considerations more explicitly in the revised manuscript and clarify that although GyrB and ParE are clearly physiological substrates of Cpe1, further work is needed to determine whether additional targets contribute to its toxic activity. We hope that the new data and expanded discussion adequately address the reviewer’s concerns.

Reviewer #2 (Christina R Bourne):

I commend the authors on their careful revision in response to previous reviewer comments, including my own. Overall I believe this is a very well executed study of high interest to multiple sub-areas in microbiology. In my opinion, the revised version is acceptable without further changes.

Reviewer #3:

This is a paper that I earlier reviewed and the authors have satisfactorily addressed all my comments

We appreciate all of the reviewers for their support of our study and the positive feedback on our revised manuscript.
